# Riluzole–Rasagiline Hybrids: Toward the Development of Multi-Target-Directed Ligands for Amyotrophic Lateral Sclerosis

**DOI:** 10.1021/acschemneuro.2c00261

**Published:** 2022-07-22

**Authors:** Claudia Albertini, Alessandra Salerno, Silvia Atzeni, Elisa Uliassi, Francesca Massenzio, Annalisa Maruca, Roberta Rocca, Marko Mecava, Filomena S. G. Silva, Débora Mena, Pedro Valente, Ana I. Duarte, Daniel Chavarria, Maicol Bissaro, Stefano Moro, Stephanie Federico, Giampiero Spalluto, Ondřej Soukup, Fernanda Borges, Stefano Alcaro, Barbara Monti, Paulo J. Oliveira, Josè C. Menéndez, Maria Laura Bolognesi

**Affiliations:** † Department of Pharmacy and Biotechnology, Alma Mater Studiorum - University of Bologna, 40126 Bologna, Italy; ‡ Dipartimento di Scienze della Salute, Università “Magna Græcia” di Catanzaro, Campus “S. Venuta”, Viale Europa, 88100 Catanzaro, Italy; § Net4Science Academic Spin-Off, Università “Magna Græcia” di Catanzaro, Campus “S. Venuta”, Viale Europa, 88100 Catanzaro, Italy; ∥ Biomedical Research Center, University Hospital Hradec Kralove, Sokolska 581, 500 05 Hradec Králové, Czech Republic; ⊥ Center for Neuroscience and Cell Biology (CNC), Centre for Innovative Biomedicine and Biotechnology (CIBB), University of Coimbra, 3004-504 Coimbra, Portugal; # Mitotag Lda, Biocant Park, 3060-197 Cantanhede, Portugal; ∇ Institute for Interdisciplinary Research (IIIUC), University of Coimbra, Casa Costa Alemão - Pólo II, Rua D. Francisco de Lemos, 3030-789 Coimbra, Portugal; ○ Research Unit for Sport and Physical Activity (CIDAF), Faculty of Sport Science and Physical Education (FCDEF-UC), University of Coimbra, 3040-248 Coimbra, Portugal; ◆ CIQUP/Department of Chemistry and Biochemistry, Faculty of Sciences, University of Porto, 4169-007 Porto, Portugal; ¶ Department of Pharmaceutical and Pharmacological Sciences, University of Padova, 35131 Padova, Italy; △ Department of Chemical and Pharmaceutical Sciences, University of Trieste, 34127 Trieste, Italy; ★ Department of Chemistry in Pharmaceutical Sciences, Organic and Medicinal Chemistry Unit, Faculty of Pharmacy, Universidad Complutense, 28040 Madrid, Spain

**Keywords:** Polypharmacology, MTDLs, ALS, benzothiazoles, MAO, riluzole, rasagiline

## Abstract

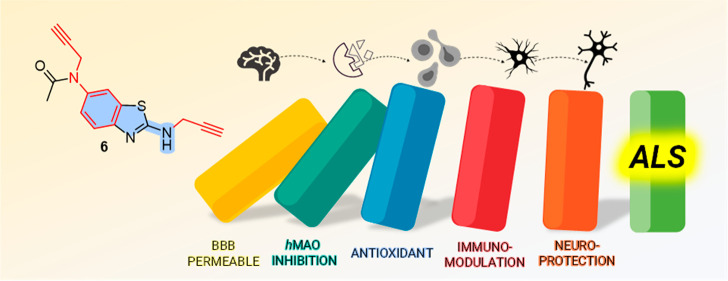

Polypharmacology is a new trend in amyotrophic lateral sclerosis (ALS) therapy and an effective way of addressing a multifactorial etiology involving excitotoxicity, mitochondrial dysfunction, oxidative stress, and microglial activation. Inspired by a reported clinical trial, we converted a riluzole (**1**)–rasagiline (**2**) combination into single-molecule multi-target-directed ligands. By a ligand-based approach, the highly structurally integrated hybrids **3**–**8** were designed and synthesized. Through a target- and phenotypic-based screening pipeline, we identified hit compound **6**. It showed monoamine oxidase A (MAO-A) inhibitory activity (IC_50_ = 6.9 μM) rationalized by *in silico* studies as well as *in vitro* brain permeability. By using neuronal and non-neuronal cell models, including ALS-patient-derived cells, we disclosed for **6** a neuroprotective/neuroinflammatory profile similar to that of the parent compounds and their combination. Furthermore, the unexpected MAO inhibitory activity of **1** (IC_50_ = 8.7 μM) might add a piece to the puzzle of its anti-ALS molecular profile.

## Introduction

**Figure 1:**
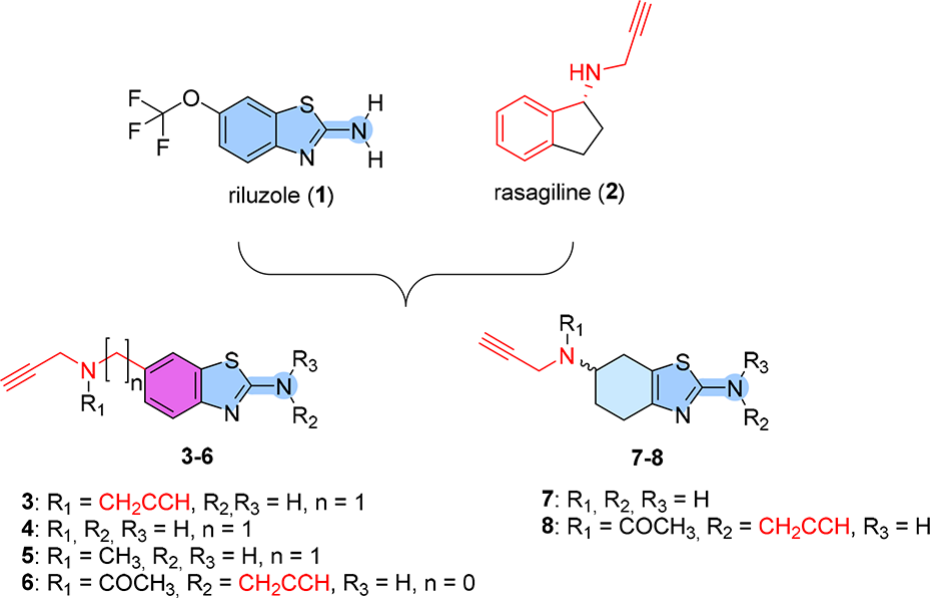
Design strategy leading to riluzole–rasagiline hybrids **3**–**8**.

Amyotrophic lateral sclerosis (ALS) is the most common neurodegenerative disease of the human neuromotor system. ALS is characterized by the progressive degeneration of motor neurons (MNs) with the consequent loss of voluntary motor activity.^1^ At present, riluzole (**1**) (Figure 1) and edaravone (Figure S1A) are the only two drugs available, although they show limited efficacy. The underestimation of ALS complexity (*e.g.*, excitotoxicity, mitochondrial dysfunction, oxidative stress, misfolded proteins, and glial cell activation), together with a still limited understanding of its etiology, may explain the current failures of both small-molecule and antisense oligonucleotide therapies.^2,3^

Given the multifactorial and complex molecular nature of ALS, polypharmacology may be considered a promising therapeutic approach, as recently reported.^4^ Our long-standing interest in the field prompted us to develop a polypharmacological approach for ALS based on multi-target-directed ligands (MTDLs).^5^ Considering the interplay between glutamate excitotoxicity, oxidative stress, and mitochondrial dysfunction,^6^ we were interested in combining the properties of **1** and rasagiline (**2**) into new MTDLs for ALS (Figure 1). Our idea was also supported by the knowledge that **1** and **2** have already shown synergistic effects in an ALS mouse model^7^ and their combination has been studied in a clinical trial.^8^

Rasagiline is an anti-Parkinson drug. It is an irreversible and selective monoamine oxidase B (MAO-B) inhibitor with additional neuroprotective/antioxidant and anti-apoptotic effects that are not dependent on MAO inhibition.^9^ Although it is not fully elucidated, riluzole acts *via* a multimodal mechanism of action that mainly involves reducing glutamatergic neurotransmission, blocking voltage-gated sodium channels, and displaying neuroprotective/antioxidant properties.^10,11^

In principle, both the riluzole–rasagiline combination and MTDLs may elicit a polypharmacological intervention. However, we envisioned that MTDLs may be favored because of the peculiar advantages of single-molecule therapy, *i.e.*, (i) better pharmacokinetics, (ii) lower risk of drug–drug interactions, and (iii) a simplified therapeutic regimen.^5^ Herein we report the design, synthesis, and biological evaluation of **3**–**8** (Figure 1) as the first riluzole–rasagiline hybrids.

## Results and Discussion

### Design and Synthesis of Hybrids **3**–**8**

To combine the beneficial properties of **1** and **2** into MTDLs for ALS, we followed a ligand-based approach (Figure 1). The chemical simplicity and fragment-like features of both **1** and **2** are likely responsible for their multiple actions and inherently promiscuous nature. Fragments are particularly promising starting points in polypharmacological drug design and can be successfully converted into MTDLs.^12^ In addition, the low molecular sizes of both parent drugs should be favorable in terms of blood–brain barrier (BBB) permeation of the resulting hybrids. By exploiting the existing structural similarities between the 1,3-benzothiazol-2-amine core of **1** and the benzene core of **2**, we designed hybrids **3**–**6**. Similarly, partially saturated analogues **7** and **8** were designed on the basis of the neuroprotective activity of pifithrin-α^13^ and dexpramipexole (Figure S1B), an investigational drug for ALS.^14^ The resulting high level of structural integration should ensure that **3**–**8** maintain the fragment-like properties of their parent compounds while potentially expanding their pharmacodynamic profile. It should be noted that the propargylamine moiety of **2** is essential not only for the MAO-B inhibitory activity but also for its neuroprotective/antioxidant and anti-apoptotic effects.^15^ Thus, one or more propargylamine moieties were introduced, directly or by a methylene spacer, at position 2 and/or 6 of the riluzole-like scaffold. Moreover, methyl (**5**) or acetyl (**6** and **8**) groups were inserted on the exocyclic nitrogen. All of the compounds reached a desirable the central nervous system multiparameter optimization (CNS-MPO)^16^ score (MPO ≥ 4; Table S1).

**Scheme 1:**
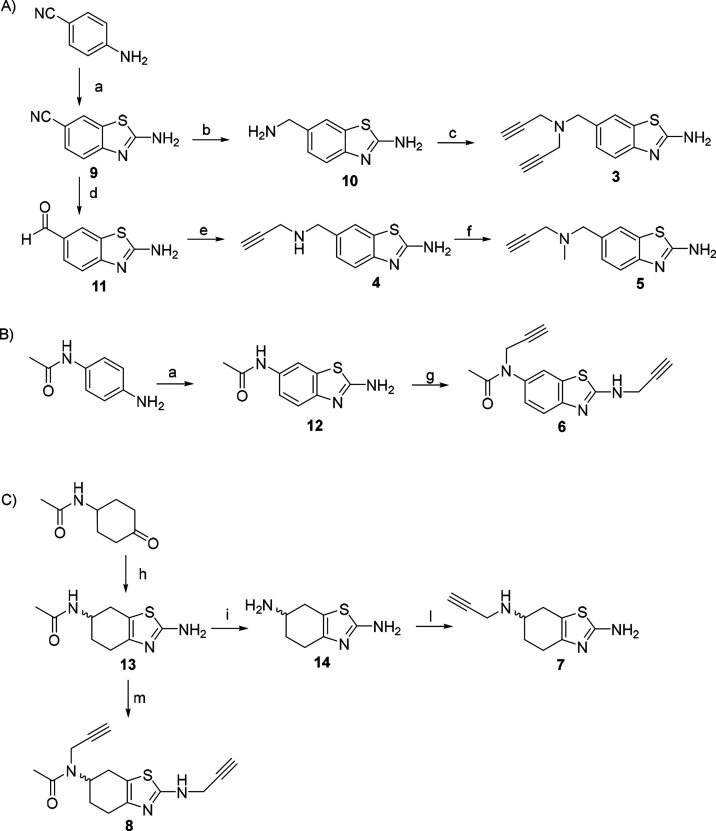
Synthesis of Riluzole–Rasagiline Hybrids **3**–**8** Reagents and conditions: (a) NH_4_SCN, Br_2_, AcOH, r.t. (55–85%); (b) NaBH_4_, BF_3_·Et_2_O, dry THF, 70 °C (42%); (c) propargyl bromide, K_2_CO_3_, ACN, r.t. (25%); (d) DIBAL-H, DCM, r.t. (76%) (e) propargylamine, NaBH_3_CN, MeOH, r.t. (60%); (f) formaldehyde (37% in MeOH), NaBH_3_CN, ACN, r.t. (49%); (g) propargyl bromide, NaH, DMF, r.t. (60%); (h) I_2_, thiourea, MeOH, MW at 100 °C, 10 min (66%); (i) HBr, 110 °C (96%); (l) propargyl bromide, K_2_CO_3_, THF, r.t. (8%), (m) propargyl bromide, NaH, DMF, 0 °C (12%).

The synthetic pathway used to synthesize hybrids **3**–**8** is depicted in Scheme 1. The 1,3-benzothiazol-2-amine core of **9** was synthesized from 4-aminobenzonitrile in the presence of ammonium thiocyanate and bromine in acetic acid (Scheme 1A) according to a variant of the Hugerschoff reaction.^17^ The subsequent reduction of the nitrile group of **9** to the amine of **10** was obtained by *in situ* production of gaseous diborane using boron trifluoride diethyl etherate and sodium borohydride, after extensive investigation. The selective monoalkylation of **10**’s aliphatic amine under classical nucleophilic substitution conditions mainly yielded *N*,*N-*dialkyl derivative **3**. Therefore, the N-monosubstituted derivative **4** was synthesized by reduction of **9**’s nitrile group to the corresponding aldehyde **11** using diisobutylaluminum hydride (DIBAL-H) and subsequent reductive amination with propargylamine. The methylation of **4**’s secondary amine was achieved by employing a reductive amination protocol with formaldehyde and sodium cyanoborohydride, giving compound **5** with high purity in good yield. Compound **12** was synthesized following the same Hugerschoff reaction described above using *N*-(4-aminophenyl)acetamide as the starting material (Scheme 1B). Treatment of **12** with a strong base and propargyl bromide afforded *N*,*N*′-dialkyl compound **6**. The unsaturated analogues **7** and **8** were synthesized from the aliphatic derivative **13**, which was prepared through a condensation reaction between cyclohexanone, iodine, and thiourea in a solvent-free reaction under microwave irradiation (Scheme 1C). Deacetylation of **13** was performed in an acidic medium, affording compound **14** in quantitative yield. N-Propargylation of the aliphatic amine was carried out under standard nucleophilic substitution conditions to give **7**. *N*,*N*′-Dipropargyl derivative **8** was obtained by exploiting a similar alkylation protocol as for the aromatic analogue.

### Biological Evaluation of Hybrids **3**–**8**

The biological profiles of hybrids **3**–**8** were evaluated by exploiting a combination of target-based and phenotypic-based screening. This was decided for two reasons: (i) the molecular mechanisms of action of both drugs are not clear yet, and (ii) both **1** and **2** are known to exert their therapeutic potential by interacting with several targets at multiple points in the ALS pathway. Clearly, this may not be achievable in a single-target-based drug screening. In addition, since ALS involves alterations in different cell types that act together to cause pathology, we developed a pipeline harnessing different insults and using neuronal and non-neuronal cell models.^18^

Considering the importance of the evaluation of BBB permeation, we first tested whether **3**–**8** are likely to cross the BBB using a parallel artificial membrane permeability assay (PAMPA) BBB assay^19^ (Table S2). In line with the MPO predictions (Table S1), all of the compounds presented permeability coefficient (*Pe*) values above 4.0, suggesting that they can cross the BBB through passive diffusion. Interestingly, the mono- and disubstitution on the exocyclic nitrogen atom modulated the compounds’ lipophilicity and improved their BBB permeation. *N*,*N*-Dipropargyl derivative **3** showed the *Pe* value, followed by the methylated compound **5**. The acetylated compound **6** exhibited a *Pe* value of 10.1 ± 0.2. Overall, the partially saturated analogues **8** (acetylated) and **7** (monopropargylated) resulted in the lowest *Pe* values (4.6 ± 0.6 and 7.7 ± 1.0, respectively).

To verify whether hybrids **3**–**8** shared the MAO inhibition properties of the parent compound **2**, we evaluated their inhibitory potencies against human recombinant *h*MAO-A and *h*MAO-B (Table S3). Autopsy studies suggest that MAO-B (and not MAO-A) is upregulated in ALS tissues.^20^ This further supports the effectiveness of MAO-B inhibitor **2** in ALS. The IC_50_ values for **3**–**8** were determined and compared with those of reference inhibitors (clorgyline and selegiline) and the parent compounds **1** and **2**. While the IC_50_ values and selectivity profile found for **2** were in agreement with literature data,^21^ surprisingly, **1** displayed selective MAO-A inhibition within the low-micromolar range (*h*MAO-A IC_50_ = 8.7 ± 0.8 μM). To the best of our knowledge, this activity has not been disclosed before. On the contrary, although structurally related, most of the derivatives (**3**, **4**, **7**, and **8**) did not reach 50% inhibition at the highest concentration tested (10 μM). Only **5** exhibited single-digit-micromolar *h*MAO inhibitory activity toward both isoforms (*h*MAO-A IC_50_ = 2.7 ± 0.4 μM; *h*MAO-B IC_50_ = 9.3 ± 1.6 μM), while compound **6** was a moderate and selective *h*MAO-A inhibitor (*h*MAO-A IC_50_ = 6.9 ± 0.5 μM). The inhibition profile toward the two isoforms may be associated with the well-known substrate permissiveness of MAO-A compared with the smaller MAO-B active site (*vide infra*).^22^ The similar IC_50_ values of **1** and **6** indicate that the propargyl groups are not fundamental pharmacophoric determinants for the observed inhibition.

**Figure 2:**
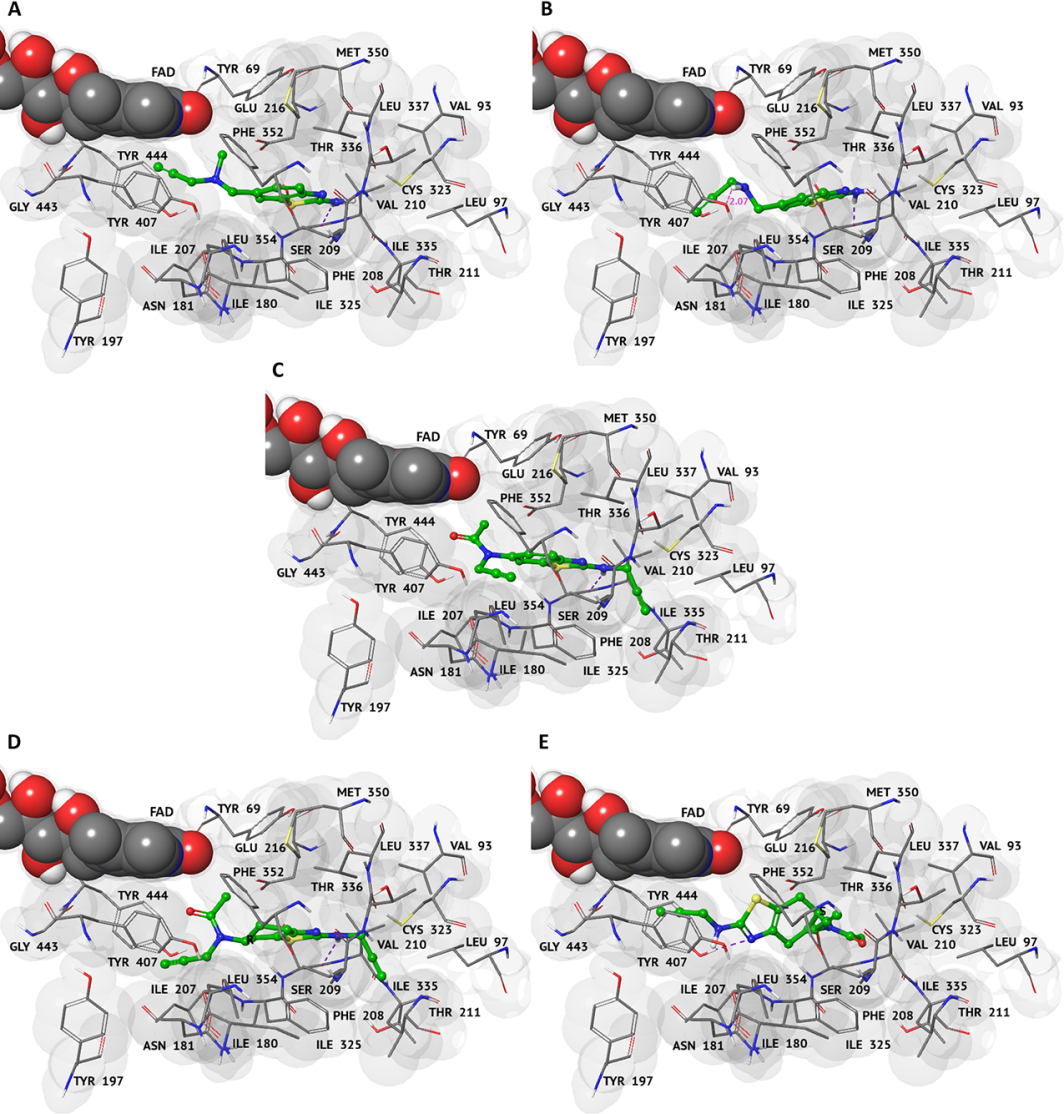
3D representations of the best complexes with *h*MAO-A. Representations were obtained after XP Glide docking simulations for compounds (A) **4**, (B) **5**, (C) **6**, and (D, E) **8** (*R* and *S* enantiomers, respectively). Ligands are shown in green carbon ball-and-stick representation. The protein is represented as a gray surface and FAD in CPK. Hydrogen-bonding interactions and distance measurements are displayed as dashed violet and magenta lines, respectively. Amino acid residues involved in the molecular interactions are shown as gray carbon sticks.

Molecular modeling studies were performed to investigate the binding modes of active (**5** and **6**) and inactive (**4** and **8**) compounds toward *h*MAOs. The parent compounds **1** and **2** (along with their *R* and *S* enantiomers) were also included. The results of the *in silico* prediction studies are in line with the IC_50_ values obtained for both isoforms *h*MAO-A and *h*MAO-B (Table S3). MAO inhibitors **5** and **6** also showed better values of interaction (with XP Glide scores of −7.11 and −7.32 kcal/mol, respectively) toward isoform A, as observed for **1** (Table S4). Docking results at the *h*MAO-A binding pocket showed that active compound **5** allows better hydrophobic interactions, whereas the secondary amine of **4** creates an electrostatic repulsion with Tyr407 (distance of 2.17 Å) (Figure 2A,B). On the other hand, the aromatic portion of **6** fits well within the *h*MAO-A hydrophobic pocket, unlike the relatively inactive compound **8** that shows reduced planarity due to the substitution of the aliphatic ring (Figure 2C–E). These results are supported also by molecular dynamics (MD) studies at *h*MAO-A (Figures S2 and S3). Both the root-mean-square deviation (RMSD) trend (Figure S2) and analysis of key interacting residues (Figure S3) pointed out the improved stability of the **5**–*h*MAO-A and **6**–*h*MAO-A complexes relative to the **4**–*h*MAO-A and **8**–*h*MAO-A complexes.

As a second target-based screening, we turned our attention to the putative casein kinase 1δ (CK1δ) inhibitory profiles of our hybrids. Recent evidence demonstrated that **1**, as well as other benzothiazole and benzimidazole derivatives, can inhibit CK1δ by binding to its hinge region and that CK1δ is linked to ALS pathological cytoplasmic aggregate of the 43 kDa transactive response DNA-binding protein (TDP-43).^23^ Upregulation of CK1δ has been observed in the spinal cord tissue and frontal cortex of ALS patients, and its inhibition attenuates MN degeneration, TDP-43 phosphorylation and accumulation, and glial reactivity both *in vitro* and *in vivo*, underscoring CK1δ as a viable therapeutic target for ALS.^24^ On the basis of the reported activity of **1**, which displays an IC_50_ of 16.1 μM against CK1δ,^25^ we preliminarily docked **3**–**6** on CK1δ (Figure S4) and evaluated their activities (Figure S5). Disappointingly, none of the compounds displayed significant inhibition when tested at concentrations up to 40 μM.

ALS has historically been considered a “neurocentric” disease that primarily affects MNs,^1^ even though recent evidence suggests that also non-neural (i.e., glial, astrocyte) and peripheral blood cells can participate in triggering MN degeneration.^18^ Peripheral cells from ALS patients (*e.g.*, fibroblasts^26^ and lymphoblasts^27^) may recapitulate peculiar pathological features and thus represent a versatile ALS cellular model easily obtainable from patients.^28^ We harnessed lymphoblasts from an ALS patient carrying the SOD1 mutation (LPS5) and a healthy donor of the same sex and age (LH5) to assess the cytotoxicities of **3**–**8** along with **1** and **2** and their combination in 1:1 ratio (**1** + **2**). Cellular viability was evaluated using the resazurin reduction assay, which estimates the metabolic activity of viable cells.^29^ The obtained data showed that hybrids **3**–**7** exhibited no cytotoxic effects up to 100 μM in both healthy LH5 (Figure S6) and mutSOD1 LPS5 (Figure S7) cell lines.

On the basis of the MAO and cytotoxicity data, we progressed **5** and **6** to the next step of our pipeline.

**Figure 3:**
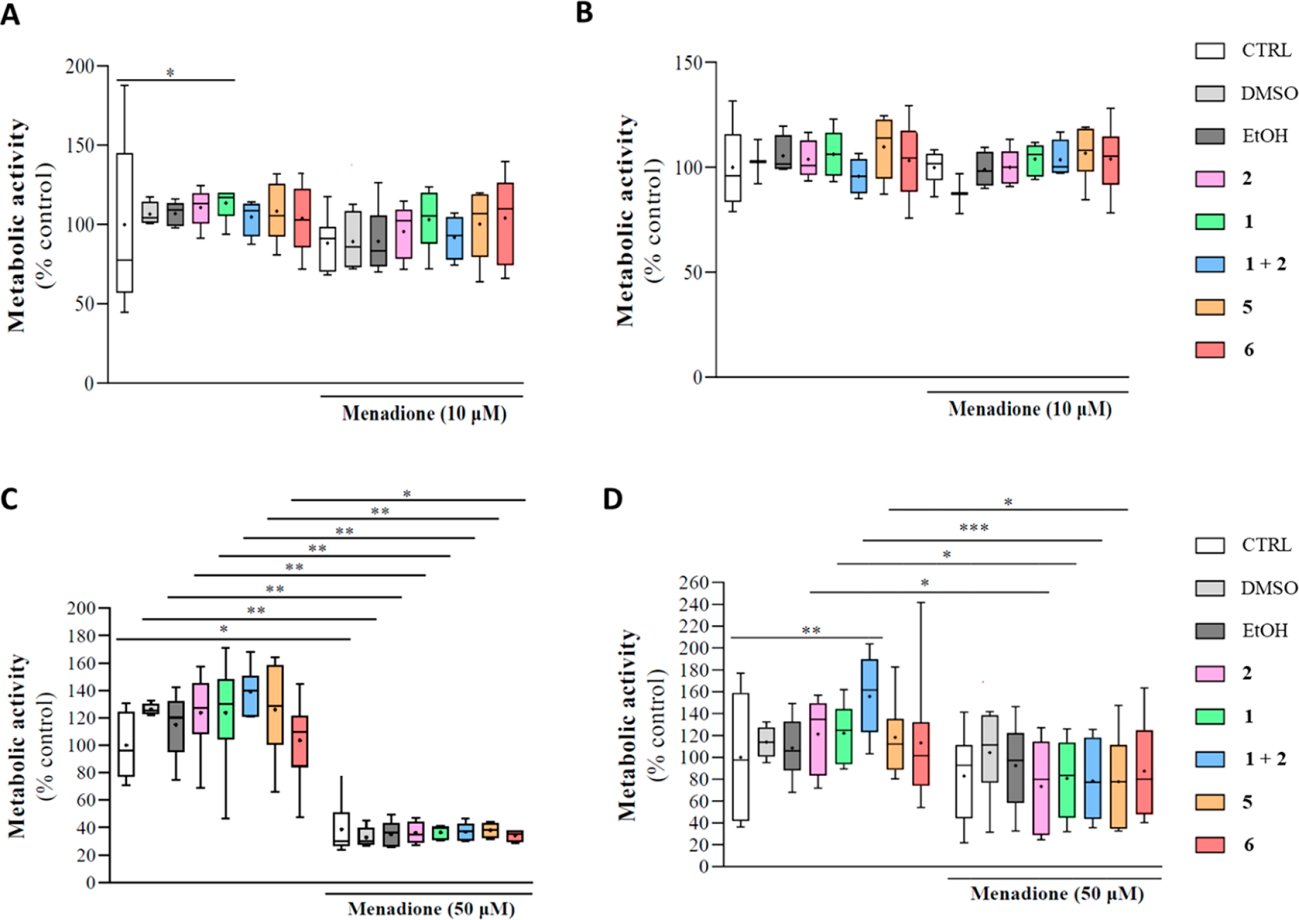
Antioxidant properties of riluzole (**1**), rasagiline (**2**), the **1** + **2** combination, and hybrids **5** and **6** after oxidative stress induction by menadione treatment in lymphoblast-derived cell lines obtained from (A, C) a healthy 46-year-old male (LH5) and (B, D) an age- and sex-matched ALS patient (LPS5). Lymphoblasts were pretreated with **1**, **2**, **1** + **2**, **5**, or **6** for 21 h, followed by a 3 h exposure to 10 or 50 μM menadione. Cell viability was evaluated by the resazurin reduction assay. Results are expressed as percentage of the control and correspond to the mean ± SEM of four to eight independent experiments run in triplicate. Statistical significance was evaluated as follows: (A) *n* = 4–8; Kruskal–Wallis (non-Gaussian), control *vs***1**, *p* = 0.03 (*); (B) no asterisk means no statistical significance compared with the control; (C) *n* = 5–6; Kruskal–Wallis, control *vs* menadione, *p* = 0.0197 (*), DMSO *vs* DMSO + menadione, *p* = 0.0012 (**), EtOH *vs* EtOH + menadione, *p* = 0.0091 (**), **2***vs***2** + menadione, *p* = 0.0039 (**), **1***vs***1** + menadione, *p* = 0.0042 (**), **1** + **2***vs***1** + **2** + menadione, *p* = 0.0013 (**), **5***vs***5** + menadione, *p* = 0.0093 (**), **6***vs***6** + menadione, *p* = 0.0185 (*); (D) *n* = 6–8; one-way ANOVA (normal), control *vs***1** + **2**, *p* = 0.0060 (**), **1***vs***1** + menadione, *p* = 0.0403 (*), **2***vs***2** + menadione, *p* = 0.0177 (*), **1** + **2***vs***1** + **2** + menadione, *p* = 0.0002 (***), **5***vs***5** + menadione, *p* = 0.0434 (*).

Propargylamines with and without MAO inhibitory properties have been found to be effective as neuroprotectants.^15,30,31^ Similarly, **1** attenuates Fe^3+^-induced lipid peroxidation,^32^ and derivatives of **1** have been shown to have antioxidant activity.^33^ On this basis, we tested **5** and **6** for their neuroprotective/antioxidant properties in LPS5 lymphoblasts, which show an increased level of reactive oxygen species (ROS).^28^ Thus, the parent compounds **1** and **2** and hybrids **5** and **6** were tested for their ability to rescue viability of LPS5 and LH5 cells exposed to an extra oxidative stress (in addition to the basal oxidative status of LPS5).^28^ This additional oxidative stress was induced by 2-methyl-1,4-naphthoquinone (menadione), which produces a semiquinone radical that reacts with O_2_ to generate ROS.^34^ Two concentrations of menadione (10 and 50 μM) that induce a cytotoxic effect were used, and the cell metabolic activity was assessed by the resazurin reduction assay (Figure 3). After insult with 10 μM menadione, LH5 cells showed a reduction of cell viability equal to 80% (Figure 3A). However, just a viability recovery trend was observed for **1**, while the **1** + **2** combination performed worse than the reference compounds individually. Of note, **5** and **6** showed a better restorative activity trend than the combination (Figure 3A). Conversely, 10 μM menadione was unable to cause a cytotoxic effect in LPS5 cells (Figure 3B). In fact, no significant effects were observed with either the parent compounds (alone or in combination) or derivatives **5** and **6** (Figure 3B). On the other hand, 50 μM menadione caused a strong decrease in LH5 cell viability (∼40%), and none of the tested compounds could rescue the induced cytotoxicity (Figure 3C). On the contrary, we detected a mild reduction of LPS5 cell viability (∼80%) using 50 μM menadione, but none of the tested compounds significantly recovered the metabolic activity (Figure 3D). These data are in line with a previous report showing that **1** counteracted the effects of H_2_O_2_ in the SH-SY5Y neuroblastoma cell line but was ineffective on the same cells carrying the familial ALS-related SOD1 mutation.^35^

**Figure 4:**
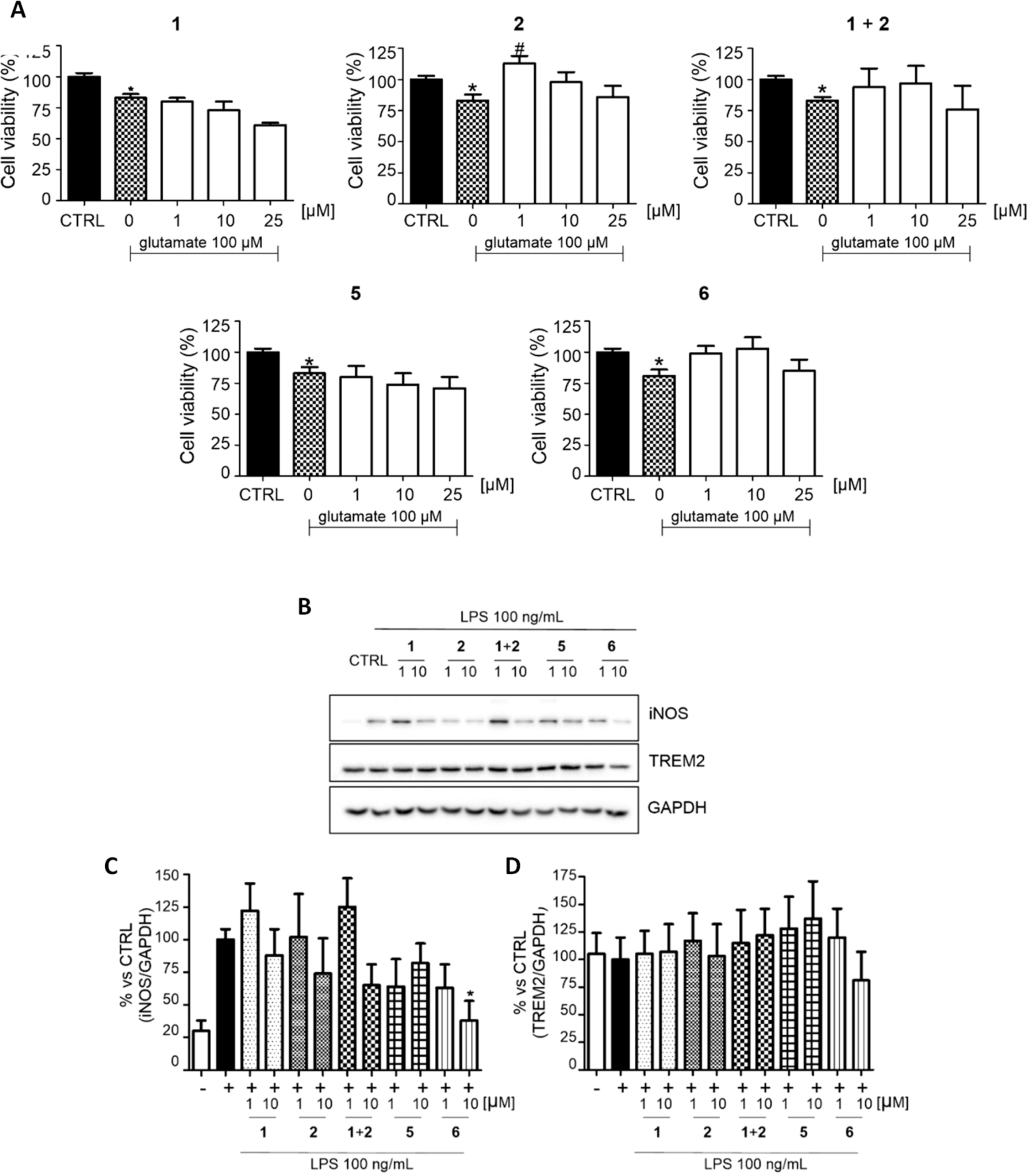
Neuroprotective and immunomodulatory effects of riluzole (**1**), rasagiline (**2**), the **1** + **2** combination, and hybrids **5** and **6**. (A) Neuroprotection in CGNs after glutamate-mediated excitotoxicity. Cell viability was evaluated through the MTT assay. (B) Immunomodulatory activity in LPS-insulted immortalized microglial cells (N9) was evaluated through Western blot analysis of biomarker expression. (C) iNOS expression. (D) TREM2 expression. GAPDH was used as the loading control. The results are expressed as the percentage of the control, and each bar is the mean ± SE of four different experiments, each run in quadruplicate. * *p* < 0.05 *vs* CTRL, *t* test; # *p* < 0.05 *vs* 0 μM, one way ANOVA, Dunnett’s multiple comparison test. No asterisk means no statistically significant difference compared to the control.

An excess of glutamate at the synaptic level is another major ALS pathophysiological mechanism.^10^ To evaluate whether **5** and **6** could maintain the ability of **1** to reduce the glutamate excitotoxicity, primary cerebellar granule neurons (CGNs) were pretreated with increasing concentrations of **5** and **6** (1, 10, 25 μM) and then exposed to 100 μM glutamate (Figure 4A). The viability of CGNs exposed to glutamate was significantly reduced to 80% compared with the control, and **1** was unable to reverse the glutamate-mediated excitotoxicity at any of the concentrations tested, in accordance with previous studies.^36^ Conversely, pretreatment with 1 μM **2** significantly reversed glutamate-induced neuronal death by increasing the viability over 100% either alone or in combination with **1**. Remarkably, **2** and derivatives featuring propargyl moieties have been shown to protect CGNs from glutamate-induced excitotoxicity,^37^ so we expected the same trend for hybrids **5** and **6**. While no significant changes were observed in cells treated with **5**, restored viability was observed for **6** at 1 and 10 μM. This result may allow us to speculate that the presence of two propargyl moieties, the carrier of the neuroprotective activity, might be responsible of the higher efficacy.

Increasing evidence indicates that neuroinflammation and microglia play important roles in ALS pathogenesis.^38^ On the basis of the evidence that **2** is able to significantly reduce the microglial neurotoxic phenotype *in vivo*,^39^ we evaluated the immunomodulatory activities of **5**, **6**, the parent compounds **1** and **2**, and their combination **1** + **2** in LPS-insulted immortalized microglial cells (N9) (Figure 4B–D). We evaluated the expression of pro-inflammatory M1 and anti-inflammatory M2 biomarkers: the M1-inducible nitric oxide synthase (iNOS) and M2-triggering receptor expressed on myeloid cells 2 (TREM2). The parent drugs **1** and **2** showed a moderate decrease of iNOS expression at 10 μM, while the **1** + **2** combination at 10 μM demonstrated more efficient reduction, suggesting a potential synergistic effect. However, significant anti-neuroinflammatory activity was shown by **6** at a concentration of 10 μM, which was able to reduce the iNOS level by about 60% (Figure 4C). With regard to the anti-inflammatory phenotype M2, all of the compounds maintained unchanged or slightly decreased the expression of TREM2 (Figure 4D).

**Figure 5:**
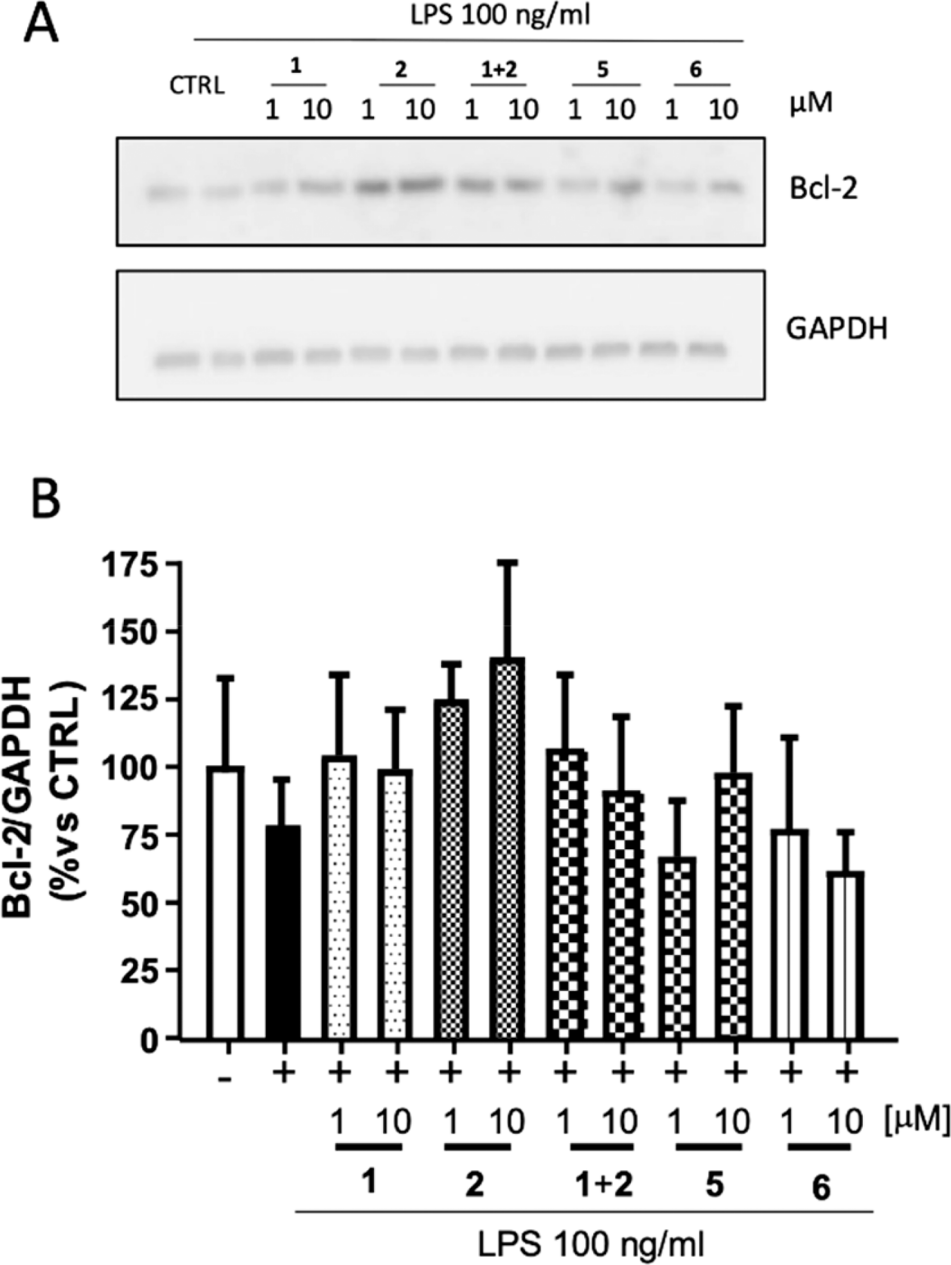
Anti-apoptotic effects of riluzole (**1**), rasagiline (**2**), and hybrids **5** and **6** in microglial cells (N9). Cells were pretreated (2 h) with compound (1 or 10 μM) and subsequently activated with LPS (100 ng/mL), and the apoptosis was evaluated through Western blot analysis of Bcl-2 expression. GAPDH was used as the loading control. Densitometric results are expressed as the percentage *vs* untreated cells (CTRL) and are the mean ± SE of four independent experiments. One way-ANOVA and Dunnett’s multiple comparison test were used.

In addition to the neuroprotective/antioxidant effects, **2** exerts anti-apoptotic actions, which appear to depend not on MAO inhibition but rather on modulating the expression of pro-apoptotic/anti-apoptotic (e.g., Bcl-2) markers.^9,21^ Similarly, **1** can modulate the activation of caspase-3 and Bcl-2.^40^ Hence, we evaluated whether **5** and **6** retain such anti-apoptotic activities in LPS-insulted microglial cells (N9). Following pretreatment with **5** and **6**, we measured Bcl-2 expression through Western blot analysis and compared it to pretreatment with **1**, **2**, or their combination **1** + **2**. Although statistical significance was not achieved under the investigated conditions, N9 cells exposed to LPS exhibited a trend of Bcl-2 expression decrease (Figure 5). As expected, **1** and **2** (more markedly) increased the level of the anti-apoptotic Bcl-2 marker, whereas the **1** + **2** combination exhibited an effect similar to that of **1**. Pretreatment of LPS-insulted N9 cells with **5** at 10 μM demonstrated a trend of recovery in Bcl-2 expression, but this was not evident for **6**.

## Conclusion

In this study, taking inspiration from the combination of **1** and **2** investigated at the clinical stage,^8^ we developed a series of hybrids **3**–**8** by combining the scaffold of **1** with that of **2**. To evaluate their multimodal profiles, we set up a pipeline based on *in vitro* brain permeability and target- (*h*MAOs and CK1δ) and phenotype-based (neuronal and non-neuronal cells, including ALS patient-derived cells) assays. All of the hybrids were predicted to be brain-permeable. *h*MAO inhibitory assays disclosed **1** to be a moderate *h*MAO-A inhibitor (IC_50_ = 8.7 ± 0.8 μM), confirming its highly promiscuous, fragmentlike nature. The MAO inhibitory profiles of **5** (*h*MAO-A IC_50_ = 2.7 ± 0.4 μM; *h*MAO-B IC_50_ = 9.3 ± 1.6 μM) and **6** (*h*MAO-A IC_50_ = 6.9 ± 0.5 μM), also rationalized by *in silico* studies, served as a basis to further progress them to phenotype-based assays. While showing no toxicity in ALS -patient-derived cells, **6** displayed an overall neuroprotective and neuroinflammatory profile in neuronal (CGN) and non-neuronal (N9 and lymphoblast) cells comparable to those of the reference compounds as well as their equimolar combination. However, no increase in the level of the Bcl-2 anti-apoptotic marker was observed.

While further optimization is required before **6** can be turned into a feasible lead for ALS, we have added new layers of information on riluzole mechanisms of action and laid the foundation for the development of ALS single-molecule polypharmacological tools. As a further remark, the applied preclinical pipeline partially based on *in vitro* patient-derived lymphoblast cells and not only on neuronal models may be exploited for further ALS drug discovery endeavors.

## Methods

Procedures for the synthesis of hybrids **3**–**8** and their characterization, MAO and CK1δ inhibitory activities, computational studies, and cytotoxicity, antioxidant, neuroprotection, immunomodulatory, and anti-apoptotic assays are included in the Supporting Information.

## Supporting Information Available

Experimental procedures for chemistry and biology, compound characterization, NMR spectra, and supplementary figures and tables (PDF)
