# Supplementary material for: Riluzole–Rasagiline Hybrids: Toward the Development of Multi-Target-Directed Ligands for Amyotrophic Lateral Sclerosis
Source: ACS Chem Neurosci. 2022 Jul 22;13(15):2252–60. doi: 10.1021/acschemneuro.2c00261 (PMC9354084; doi:10.1021/acschemneuro.2c00261)
Supplement: Supplementary file 1 — cn2c00261_si_001.pdf [file cn2c00261_si_001.pdf]

Riluzole-Rasagiline Hybrids: Towards the Development of Multi-Target-Directed Ligands for Amyotrophic Lateral Sclerosis

Claudia Albertini,[(a)‡] Alessandra Salerno,[(a)‡] Silvia Atzeni,[(a)] Elisa Uliassi,[(a)] Francesca Massenzio,[(a)] Annalisa Maruca,[(b,c)] Roberta Rocca,[(b,c)] Marko Mecava,[(d)] Filomena S. G. Silva,[(e,f)] Débora Mena,[(e,g)] Pedro Valente, [(e,h)] Ana I. Duarte,[(e,g)] Daniel Chavarria,[(i)] Maicol Bissaro,[(l)] Stefano Moro,[(l)] Stephanie Federico,[(m)] Giampiero Spalluto,[(m)] Ondřej Soukup,[(d)] Fernanda Borges,[(i)] Stefano Alcaro,[(b,c)] Barbara Monti,[(a)] Paulo J. Oliveira,[(e,g)] Josè C. Menéndez,[(n)] Maria Laura Bolognesi[(a)*]

[*]Corresponding author: Maria Laura Bolognesi; Email: marialaura.bolognesi@unibo.it

Supporting Information

Table of contents

Figure S1. Structure of drugs currently on the market for ALS treatment., riluzole's saturated analogs dexpramipexole and pifithrin-α. ……………………………………………………………………..S3

Table S1. Central Nervous System Multiparameter Optimization (CNS MPO) function of compounds **3-8** compared with riluzole (**1**)……………………………………………………………………………S3

Table S2. *In vitro* permeability coefficient (P_e) values with related predictive penetrations into the CNS of commercial drugs and compounds **3-8**……………………………………………………………………S4

Table S3. *h*MAO-A and *h*MAO-B inhibitory activities of compounds **3-8** and reference compounds………S5

Table S4. XP Glide Score (G-Score) value of the best synthesized derivatives by docking simulation on both isoforms *h*MAO-a and *h*MAO-B....……………………………………………………………………S6

Figure S2 Trend of the Root Mean Square deviation (RMSd) during MD simulation for compounds **4-6** and both enantiomers of **8**. ……………………………………………………………………………..S7

Figure S3. Analysis of ligand atom interactions with *h*MAO-A residues after MD simulation………………S8

Figure S4. Molecular docking studies of compounds **3-5** toward CK-1δ in comparison to riluzole (**1**) ………S9

Figure S5. CK-1δ residual activity after treatment with compounds **3-6** at 40 µM…………………………..S9

Figure S6. Cytotoxicity evaluation of compounds **3-8** in comparison with riluzole (**1**), rasagiline (**2**) and their combination (**1**+**2**) in a lymphoblast cell line (LH5) obtained from healthy male …………………………S10

Figure S7. Cytotoxicity evaluation of compounds **3-8** in comparison with riluzole (**1**), rasagiline (**2**) and their combination (**1**+**2**) in a lymphoblast cell line (LPS5) obtained from an ALS patient carrying the SOD1 mutation.…………………………………………………………………………………………S11

Figure S8-S19. Representative compounds ^{1}H NMR and ^{13}C NMR Spectra……………….……….......S12-S17

MATERIAL AND METHODS

Chemistry……………………………………………………………………………….…….......S18-S23

Molecular modelling studies of *h*MAO inhibition……………………………..……….……......S23-S24

PAMPA assay...………………………..…………………………….……..……..............................S24

Evaluation of Human Monoamine Oxidase (*h*MAO) Inhibitory Activity.……………………......S24-S25

Cell viability in lymphoblasts……………………………..………..……..S25

Antioxidant activity in human lymphoblasts………………………………..S26

Neuroprotective effect on glutamate-mediated excitotoxicity in CGNs ……………………................S26

Immunomodulation in N9 cell line…………………………………….…………………….................S26

Antiapoptotic effects in N9 cell line…………………………………….………………........................S27

Molecular Docking at CK-1δ……………………………………….…………………….................S27

CK-1δ activity assay……………………………………………………….………………............S27-S28

Bibliography......………………………………………………….………………………..…….…..S29

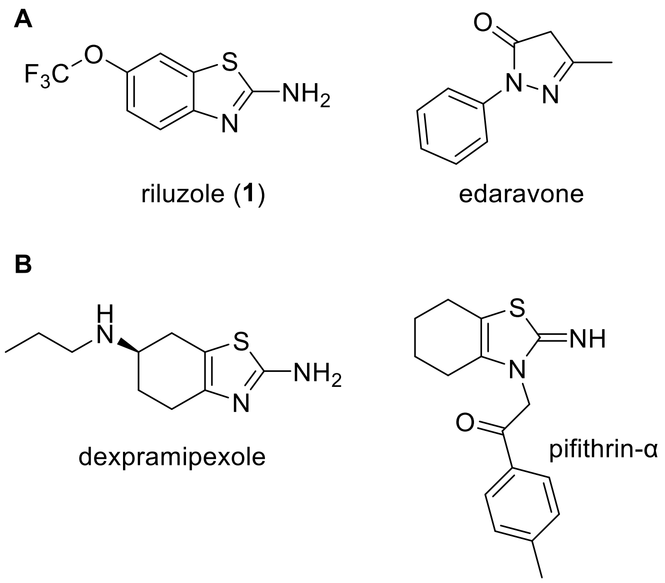

Figure S1. A) Drugs currently on the market for ALS treatment. B) Riluzole's saturated analogues dexpramipexole and pifithrin-α.

Table S1. Central Nervous System Multiparameter Optimization (CNS MPO) function[1] of compounds **3-8** in comparison to riluzole (**1**).

Compound	MW	cLogP	cLogD	HBD	TPSA	PKa	CNS MPO Score
**1**	234.2	3.61	3.40	2	48.14	4.57	
(riluzole)							4.5
**2**	171.24	2.65	1.02	1	16,61	6.95	
(rasagiline)							4.8
**3**	255.34	2.10	2.18	2	42.15	7.05	5.4
**4**	217.29	1.51	0.67	3	50.94	8.16	5.2
**5**	231.32	1.98	1.46	2	42.15	7.89	5.5
**6**	283.35	2.28	1.83	1	45.23	3.29	5.8
**7**	207.30	1.08	-0.44	3	45.23	3.29	5.3
**8**	287.38	1.79	1.25	1	50.94	8.95	5.3

The physicochemical parameters were computed by FAF-Drugs4 and Marvin Sketch.[2]

Table S2. *In vitro* permeability coefficient (P_e) values with related predictive penetrations into the CNS of commercial drugs and compounds **3-8.**[a]

**Compound**	**Pe ($\times 10^{-6}$ cm s^{-1})**[a]	**CNS prediction** [b]
**3**	24.89 ± 0.25	CNS +
**4**	9.3 ± 0.1	CNS +
**5**	22.70 ± 0.6	CNS +
**6**	10.15 ± 0.25	CNS +
**7**	7.7 ± 1.0	CNS +
**8**	4.6 ± 0.6	CNS +
furosemide	0.19 ± 0.07	CNS -
chlorothiazide	1.14 ± 0.53	CNS -
cefuroxime	0.62 ± 0.16	CNS -
donepezil	21.49 ± 2.05	CNS +
rivastigmine	20.00 ± 2.07	CNS +
tacrine	5.96 ± 0.59	CNS +

[a]*Pe*± SEM (n = 3 for standards drugs, n = 2 for novel compounds); [b]CNS +: high BBB permeation predicted, *Pe* ($\times 10^{-6}$ cm s^{-1}) > 4.0; CNS -: low BBB permeation predicted, *Pe* ($\times 10^{-6}$ cm s^{-1}) < 2.0.

Table S3. *h*MAO-A and *h*MAO-B inhibitory activities of compounds **3-8** and reference compounds.

Compound	IC_{50} (µM)		SI[b]
	*h*MAO-A	*h*MAO-B	
**3**	-[a]	-[a]	-
**4**	-[a]	-[a]	-
**5**	2.73 ± 0.39	9.31 ± 1.57	0.29
**6**	6.88 ± 0.46	-[a]	< 0.69 [c]
**7**	-[a]	-[a]	-
**8**	-[a]	-[a]	-
riluzole (**1**)	8.71 ± 0.81	-[a]	< 0.87 [c]
rasagiline (**2**)	2.27 ± 0.08	0.109 ± 0.014	20.9
selegiline	23.0 ± 4.2	0.0526± 0.0065	436.1
clorgyline	0.00193 ± 0.00022	2.89 ± 0.53	0.00067

[a]Compounds did not reach 50% inhibition at the highest compound concentration tested (10 µM); [b]SI: *h*MAO-B selectivity index = IC_{50} (*h*MAO-A)/IC_{50} (*h*MAO-B); [c]Values obtained under the assumption that the corresponding IC_{50} value against *h*MAO-A or *h*MAO-B is the highest concentration tested (10 µM).

Table S4. XP Glide Score (G-Score) value of the best synthesized derivatives by docking simulation on both isoforms *h*MAO-A and *h*MAO-B.

Compound	XP G-Score	
	*h*MAO-A	*h*MAO-B
**1**	-6.10	-5.46
**(*R*)-2**	-6.28	-9.15
**(*S*)-2**	-6.81	-6.70
**4**	-5.97	-5.58
**5**	-7.11	-7.49
**6**	-7.32	-4.84
**(*R*)-8**	-5.09	-6.42
**(*S*)-8**	-5.31	-6.34

[a] G-Scores values are expressed in kcal/mol.

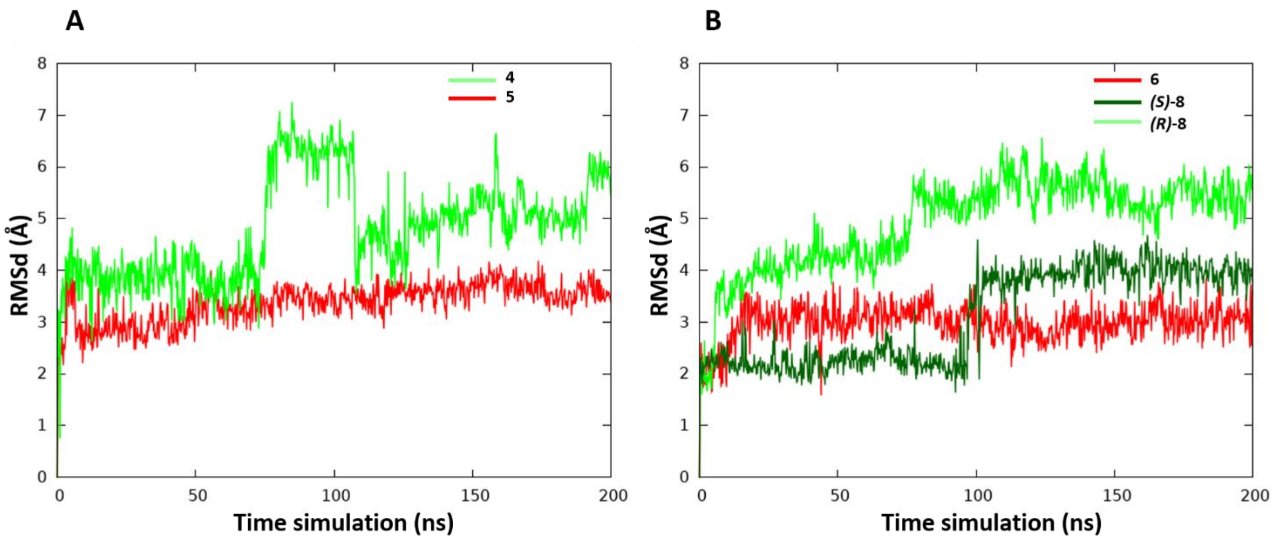

Figure S2. Trend of the Root Mean Square deviation (RMSd) during 200 *ns* of MD simulation for *h*MAO-A-compound complexes: (A) **4** and **5** (red and light-green lines, respectively), (B) **6**, (*S*)-**8** and (*R*)-**8** (red, light-green, and dark-green lines, respectively). RMSd values were calculated on ligand heavy atoms, after the protein-ligand complex has been aligned on the protein backbone of the first frame. Active compounds **5** and **6** (red lines in panels A and B), after an initial adjustment of their poses in the first 20 *ns* of simulation, maintained a stable binding mode with an average RMSd value of 3.36 Å and 2.97 Å, respectively. **4** and (*R*)-**8** (light-green lines in panels A and B) showed a progressive and considerable change in their binding modes, with an average RMSd value of 4.78 Å and 4.91 Å, respectively. (*S*)-**8** (dark-green line in panel B) exhibited a good conservation of the starting pose in the first 100 *ns*, to substantially change in the second half of MD simulation.

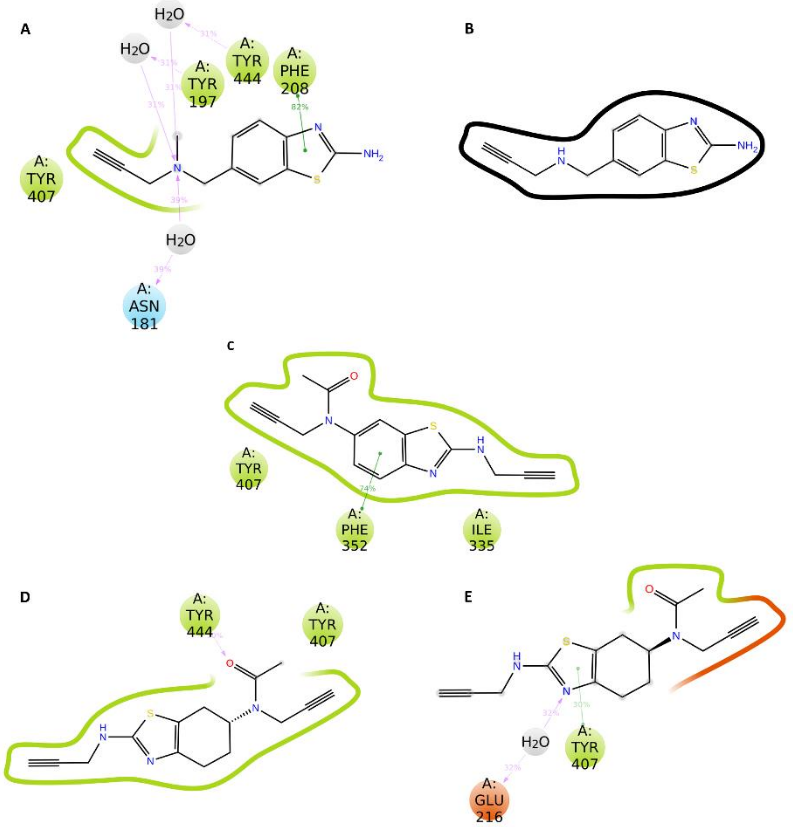

Figure S3. Ligand atom interactions with *h*MAO-A residues after MD simulation for compounds: (A) **5**, (B) **4**, (C) **6**, (D-E) **8** (*R* and *S* enantiomers, respectively). Only significant interactions, *i.e.*, occurring more than 30.0% of the trajectory simulation time (200 *ns*), were shown. (A) Tertiary amine of **5** allowed the establishment of a consistent network of water bridges with residues Asn181, Tyr197 and Tyr444, a π–π stacking with Phe208, and several hydrophobic interactions; (B) inactive compound **4** did not establish any significant interactions; (C) the binding mode of **6** is stabilized by a stacking interaction with Phe352; (D-E) the higher flexibility of **8** (both *R* and *S* enantiomers) resulted in weaker stacking and hydrophobic interactions, with consequent lower stability and affinity.

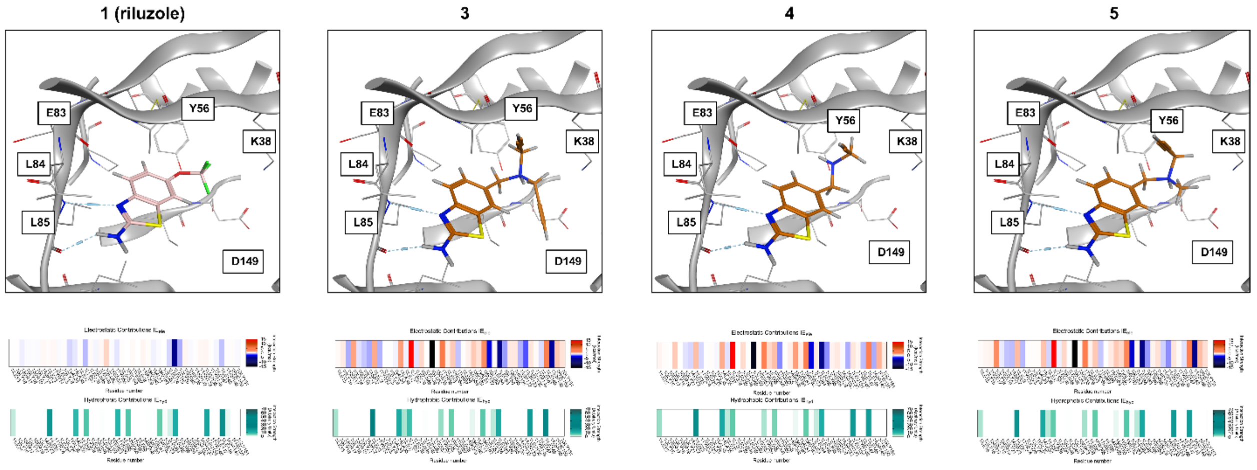

Figure S4. Molecular docking studies of compounds **3-5** in complex with the protein kinase CK-1δ, compared to the reference CK-1δ inhibitor riluzole (**1**).

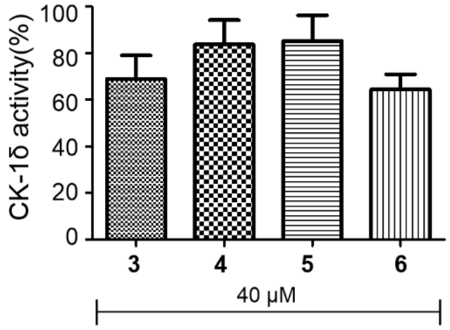

Compound	residual CK-1δ activity (%)	SD
**3**	64.4	12.9
**4**	69.0	19.9
**5**	83.7	20.7
**6**	85.2	19.1

Figure S5. CK-1δ residual activity after treatment with compounds **3-6** at 40 μM.

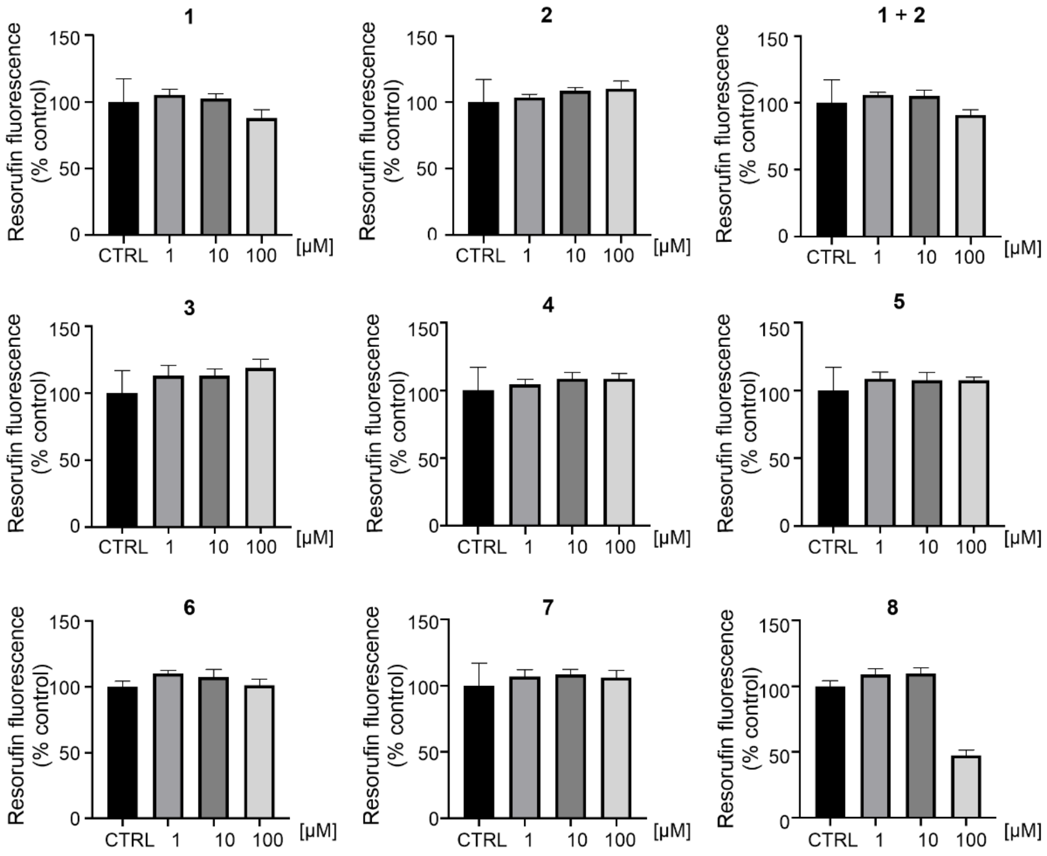

Figure S6. Cell viability in healthy lymphoblasts. Cell viability was determined by a test based on resazurin reduction assay in a lymphoblast cell line obtained from a 46-years-old healthy male (healthy). Lymphoblasts were treated with compounds **3-8** at different concentrations (1, 10, 100 µM) in comparison with control (DMSO 0.1%). Results are expressed as percentages of controls and are the mean ± SE of at least 3 independent experiments, each run in triplicate.

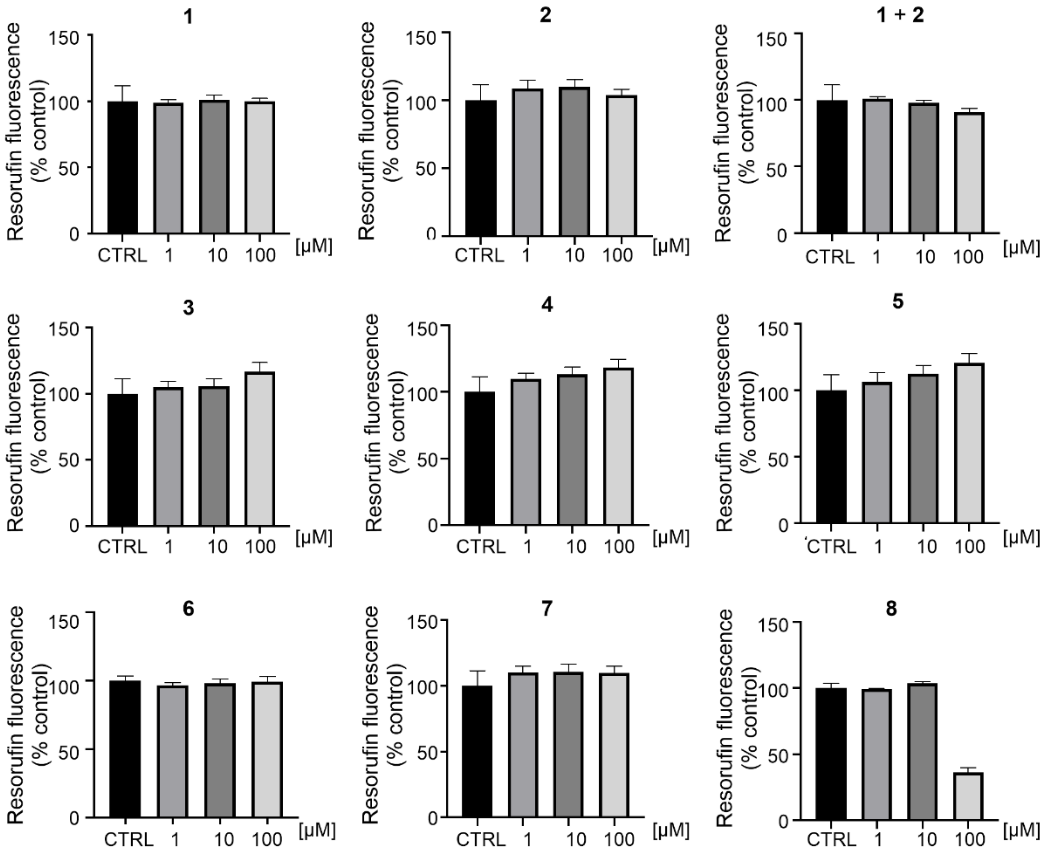

Figure S7. Cell viability in mutSOD1 lymphoblasts. Cell viability was determined by a test based on resazurin reduction assay in lymphoblast cell lines from one ALS patient with 46-years-old with mutant SOD1 (mutSOD1). Lymphoblasts were treated with increasing concentrations of compounds **3**-**8** (1, 10, 100 µM) in comparison with control (DMSO 0.1%). Results are expressed as percentages of controls and are the mean ± SE of at least 3 independent experiments, each run in triplicate.

Figure S8. ^{1}H NMR ($(CD_3)_2CO$, 250 MHz) of compound **3**

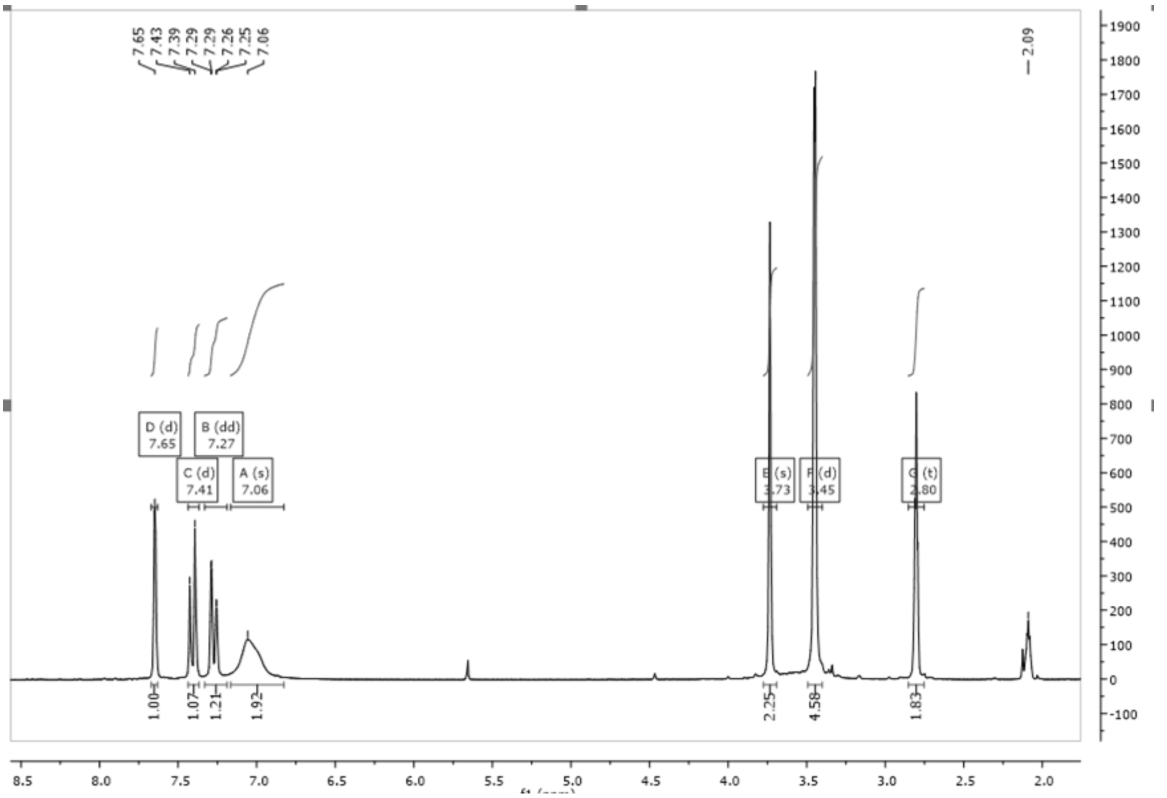

Figure S9. ^{13}C NMR ($(CD_3)_2CO$, 250 MHz) of compound **3**

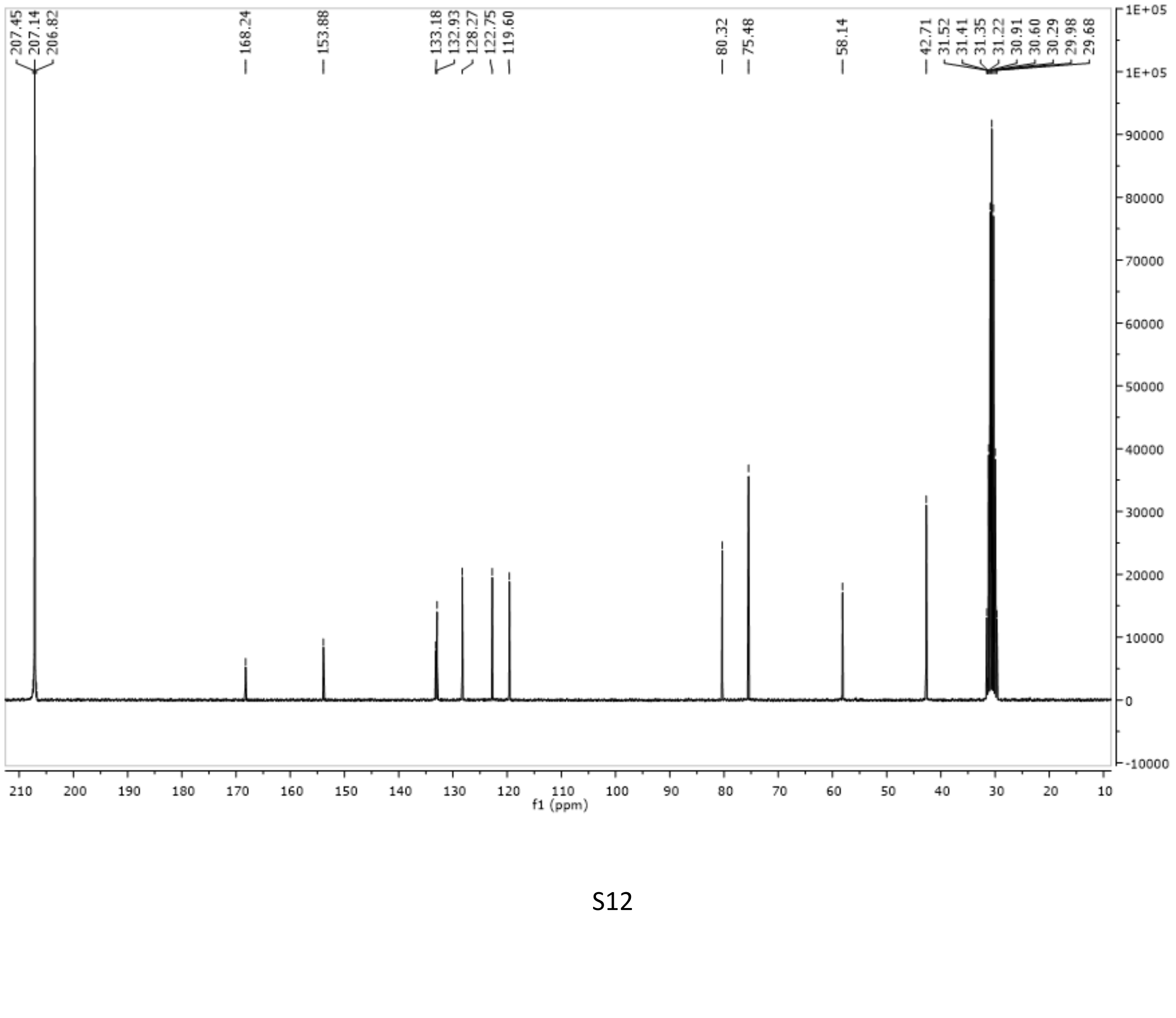

Figure S10. ^{1}H NMR (CD_3OD, 250 MHz) of compound **4**

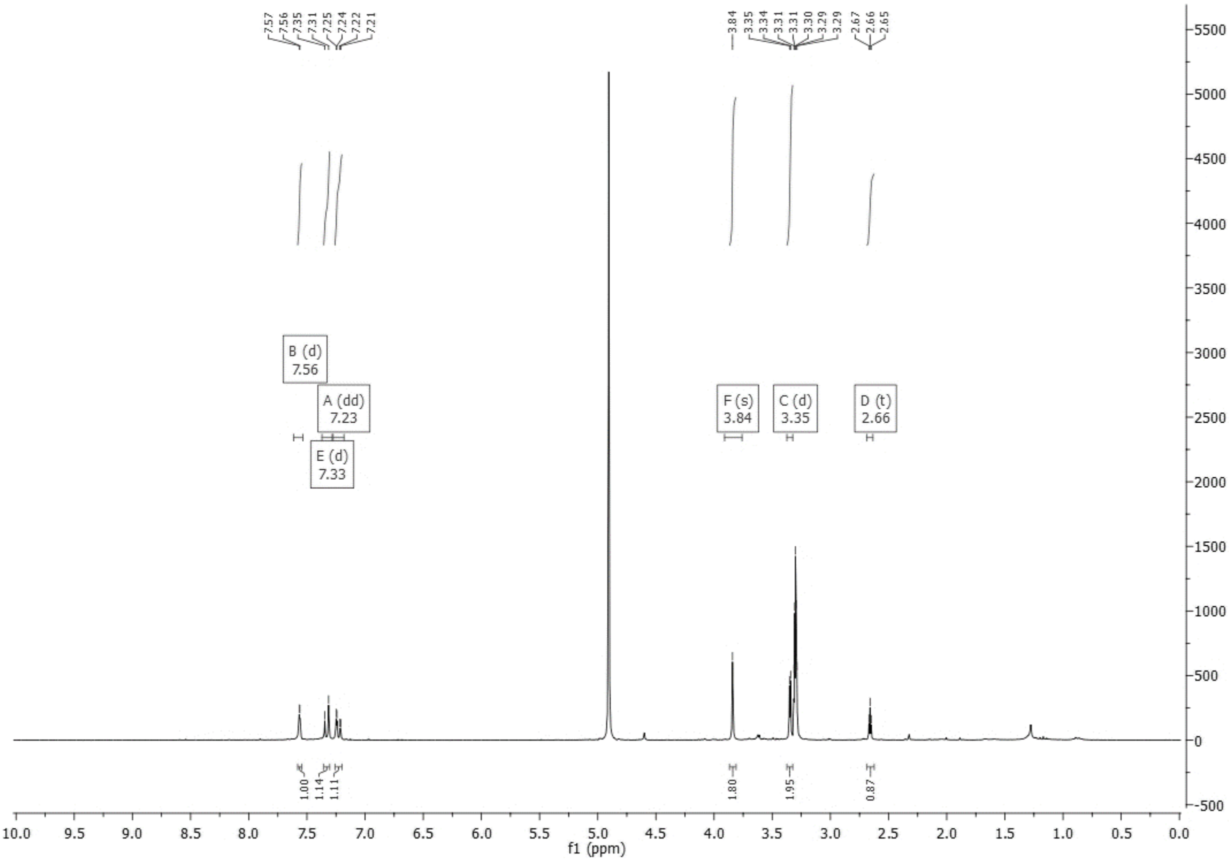

Figure S11. ^{13}C NMR ($(CD_3)_2CO$, 250 MHz) of compound **4**

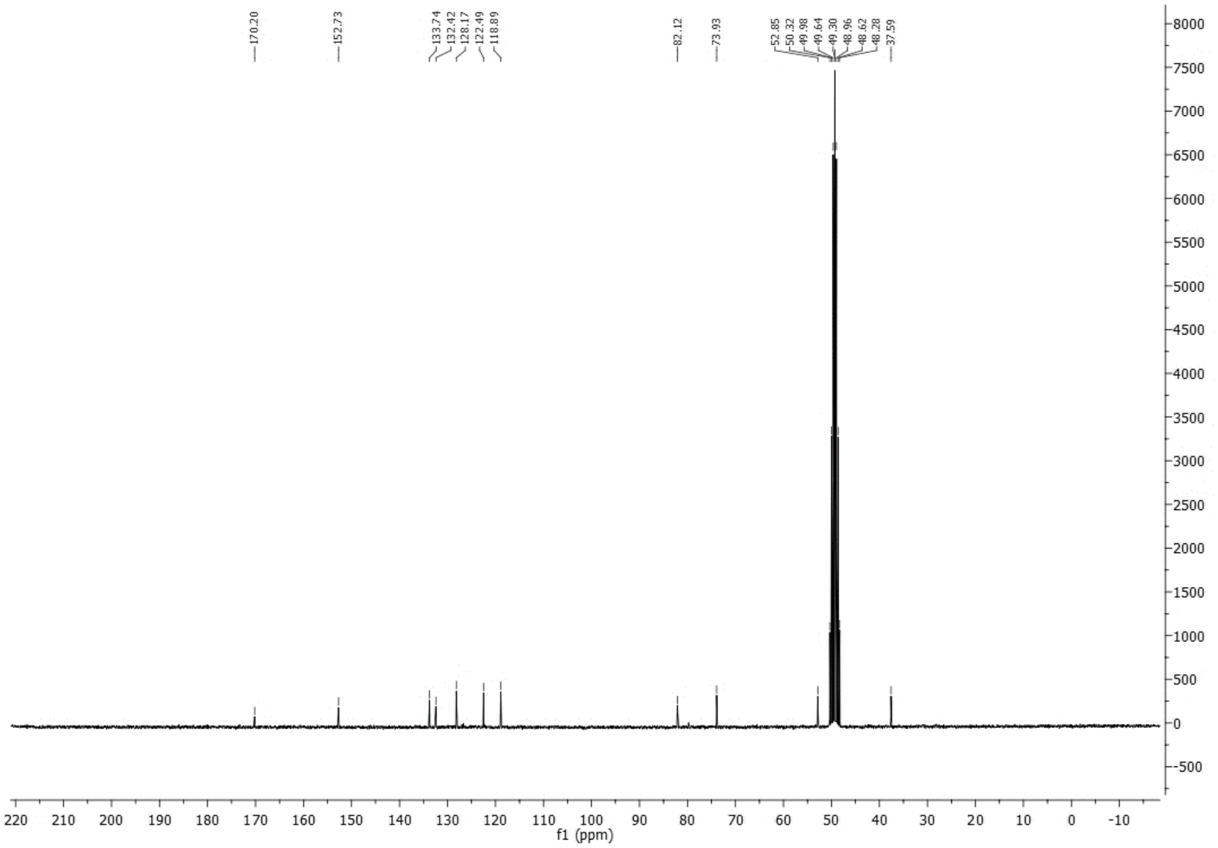

Figure S12. ^{1}H NMR (CD_3OD, 250 MHz) of compound **5**

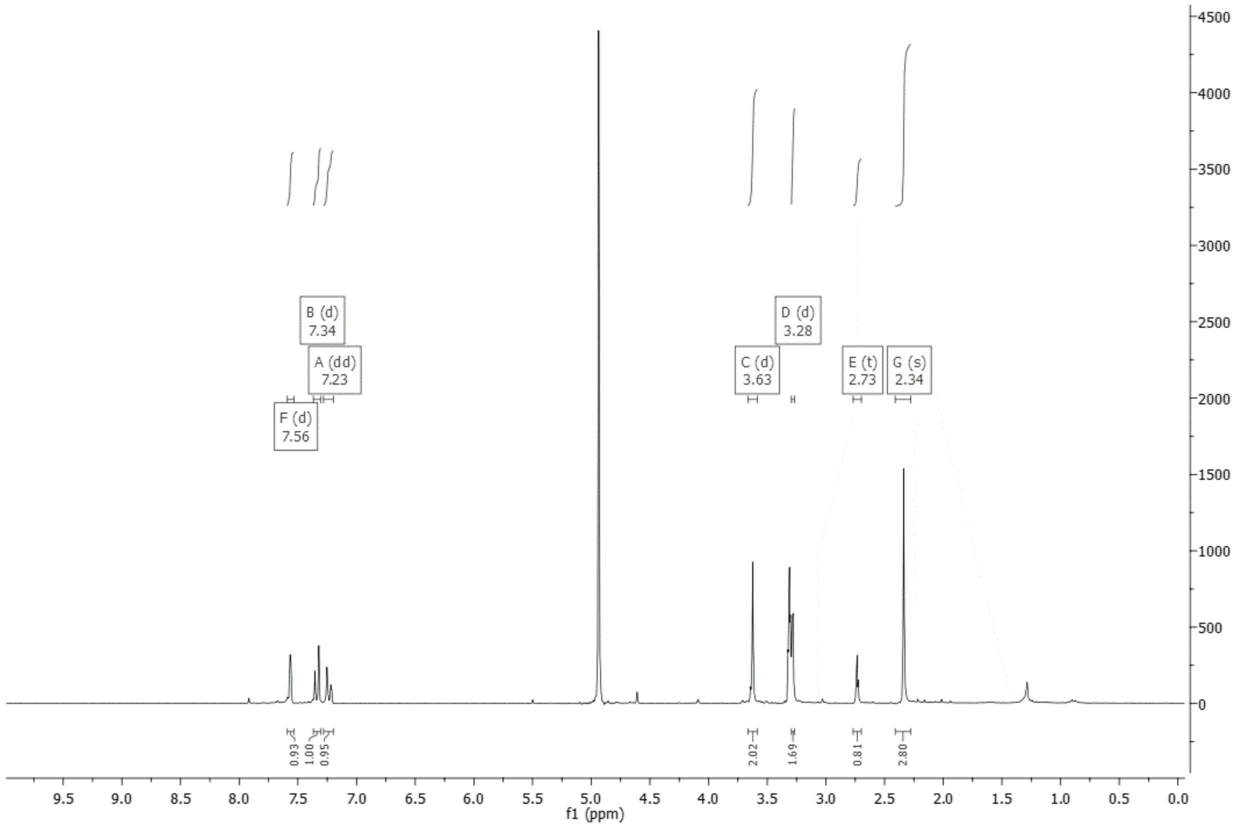

Figure S13. ^{13}C NMR (CD_3OD, 63MHz) of compound **5**

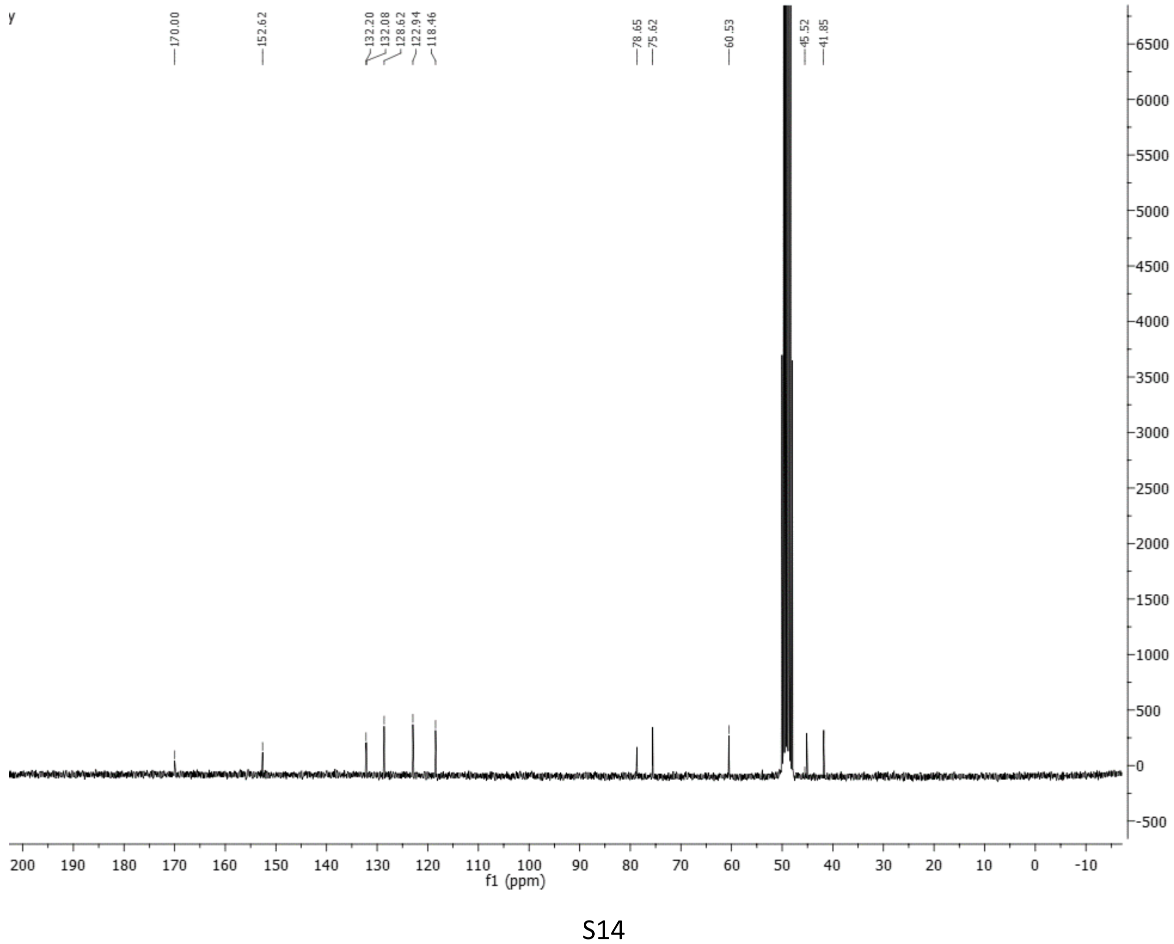

Figure S14. ^{1}H NMR (CD_3OD, 250 MHz) of compound **6**

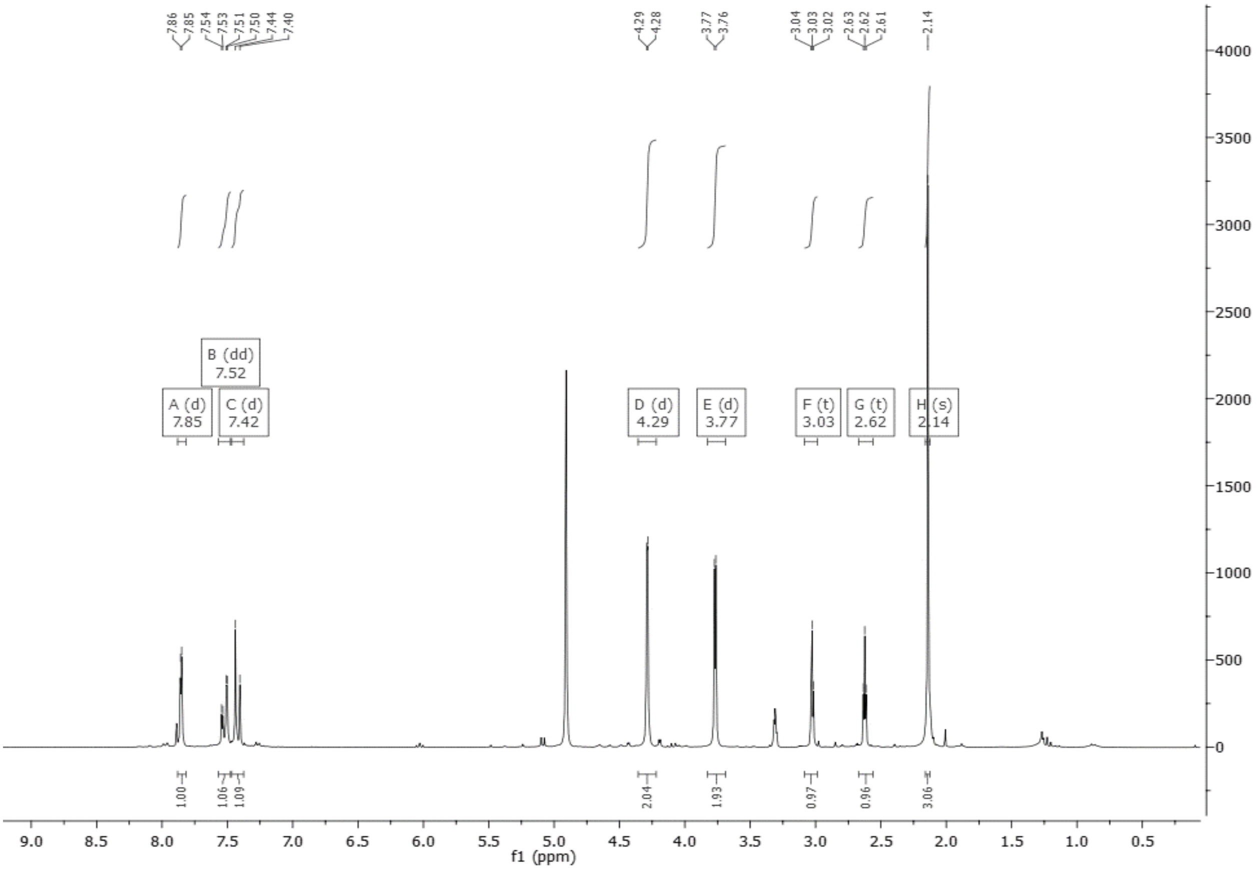

Figure S15. ^{13}C NMR (CD_3OD, 63MHz) of compound **6**

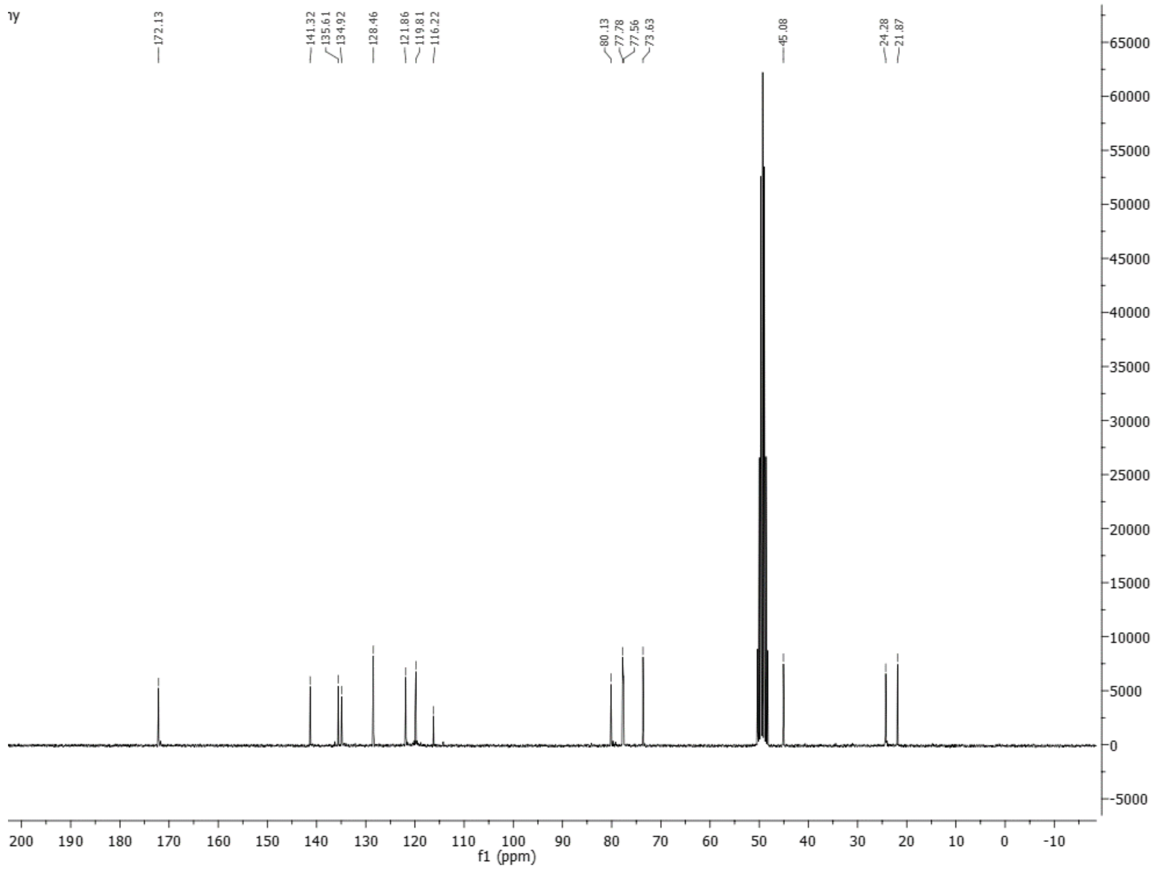

Figure S16. ^1H NMR (CD_3OD, 250 MHz) of compound **7**

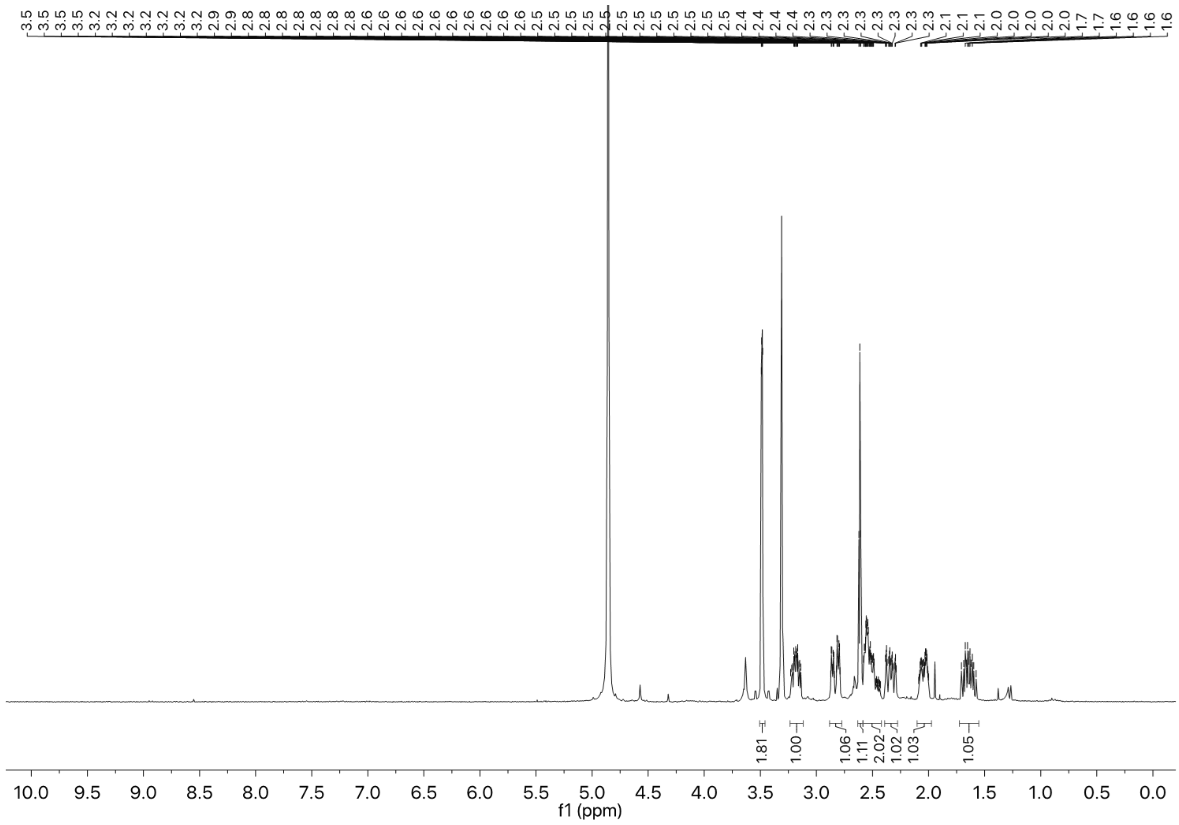

Figure S17. ^{13}C NMR (CD_3OD, 63MHz) of compound **8**

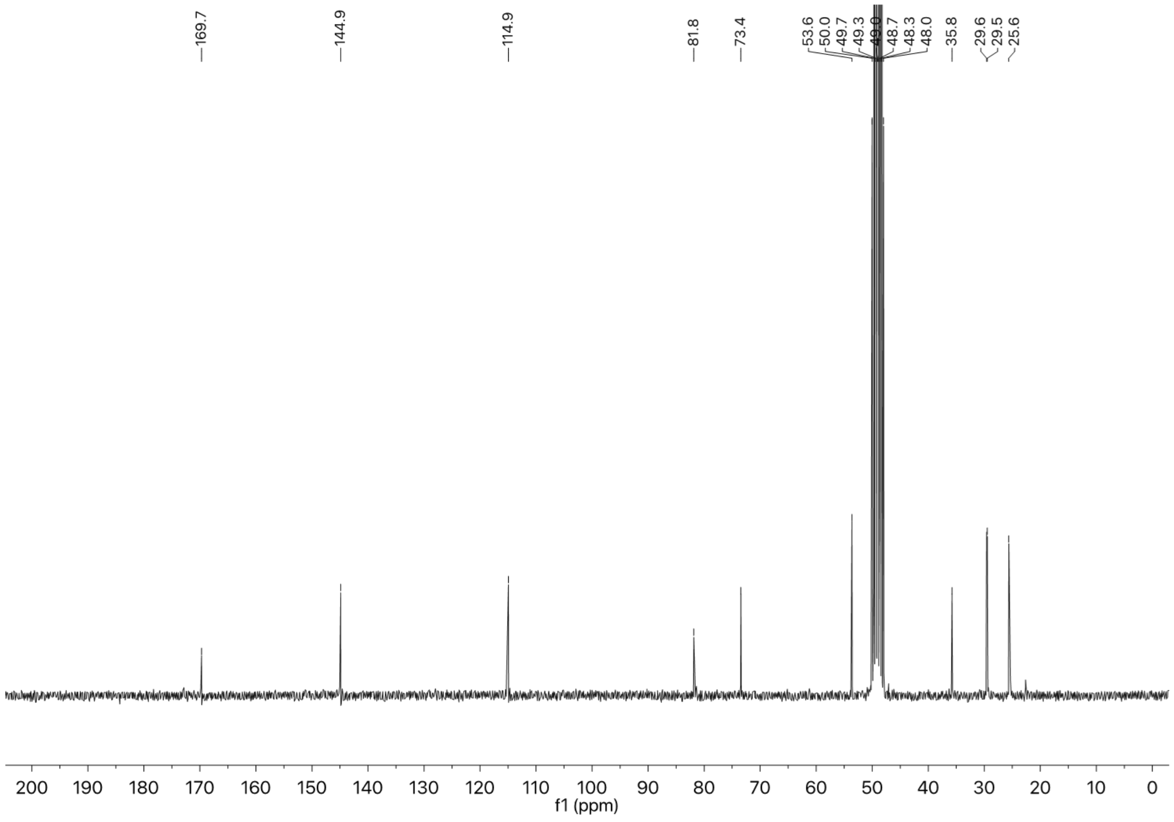

Figure S18. ^{1}H NMR (CD_3OD, 250 MHz) of compound **8**

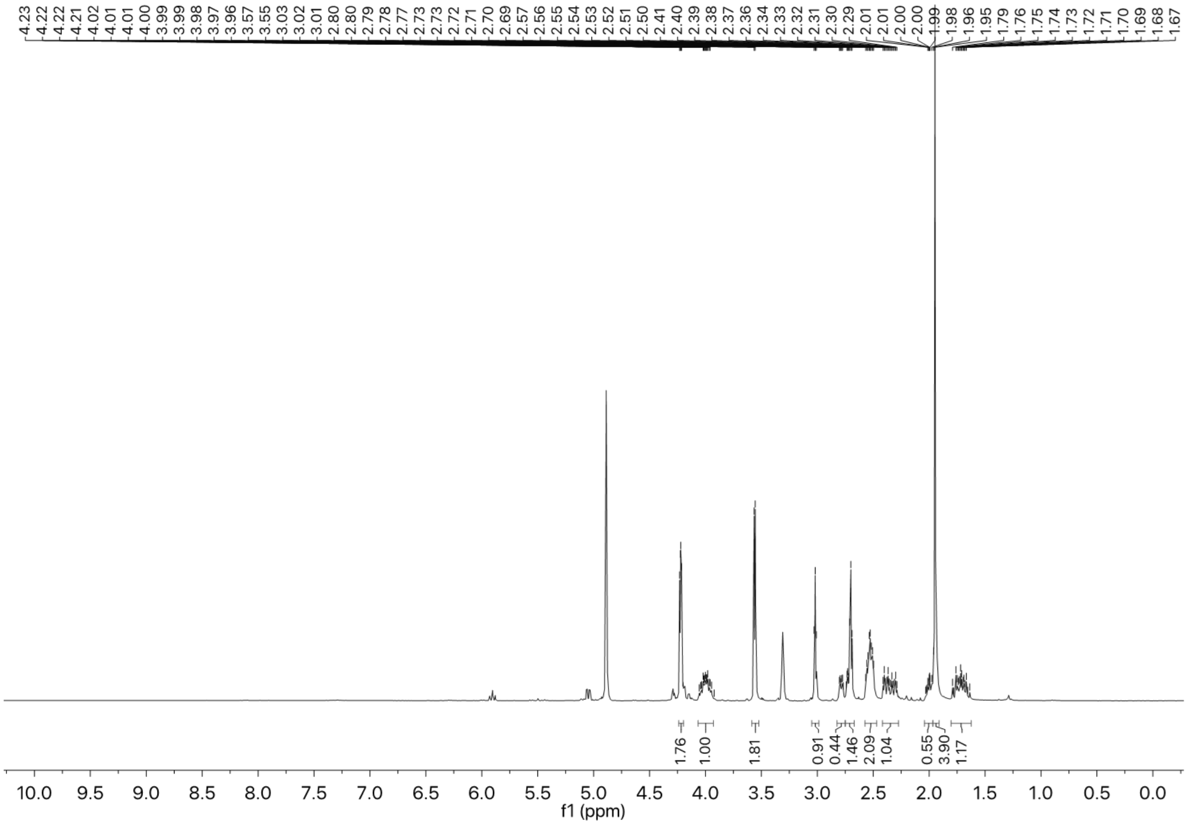

Figure S19. ^{13}C NMR (CD_3OD, 63MHz) of compound **8**

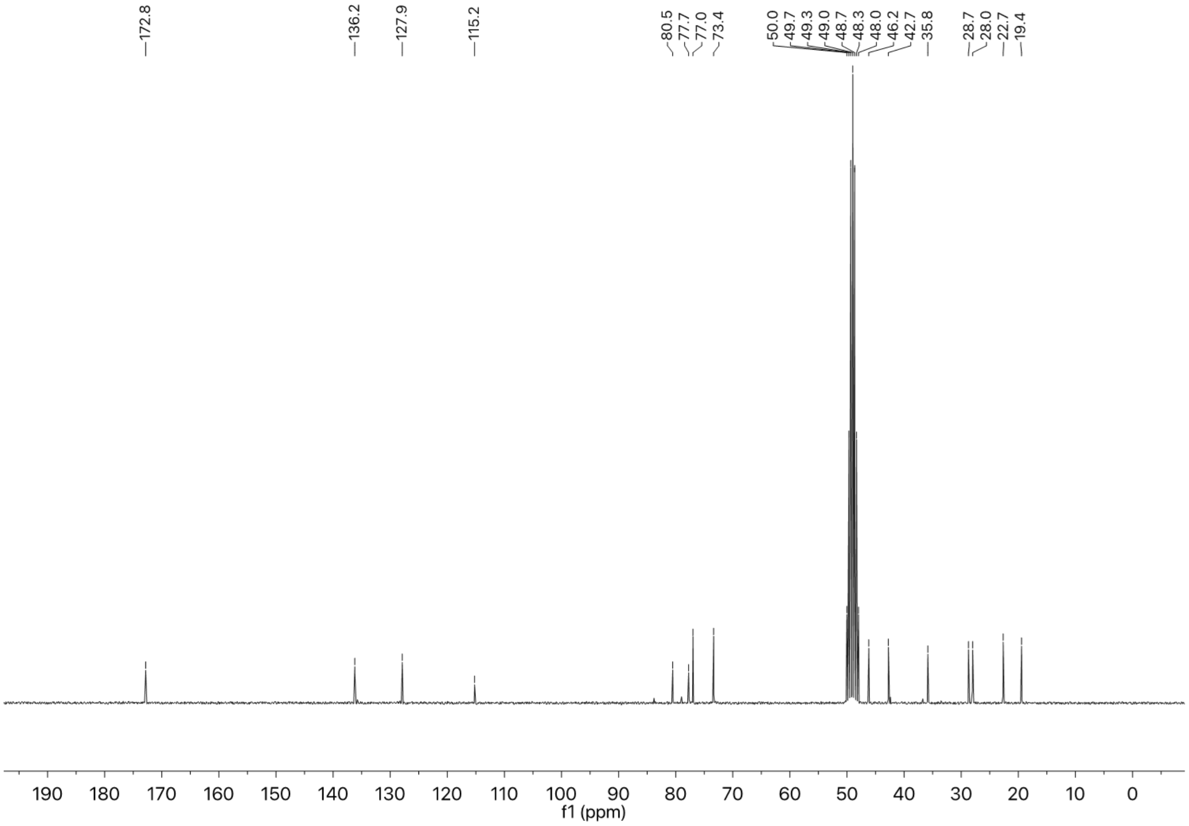

MATERIAL AND METHODS

Chemistry. All reagents (Merk, Madrid, Spain; Fischer Chemical, Madrid, Spain; Alpha Aesar, Kändel, Germany) and solvents (Scharlau, Barcelona, Spain; Fischer Chemical, Madrid, Spain) were of commercial quality and used without further purification (with exception of boron trifluoride diethyl etherate, which was freshly distilled before the use). Reactions were monitored by thin layer chromatography (TLC) on aluminum plates coated with silica gel and fluorescent indicator (layer: 0.20 mm silica gel 60 with a fluorescent indicator UV254, from Merck, Madrid, Spain). Microwave-assisted reactions were performed on a CEM Discover focused microwave reactor NMR spectroscopic data were recorded using a Bruker Avance 250 spectrometer (Bruker, Rivas-Vaciamadrid, Spain) operating at 250 MHz for ^{1}H-NMR and 63 MHz for ^{13}C-NMR and maintained by the NMR facility of Universidad Complutense (Resonancia Magnética Nuclear Unit, Madrid, Spain); chemical shifts are given in δ parts per million (ppm) and coupling constants in Hertz. Melting points (mp) were determined using a Stuart Scientific apparatus, SMP3 Model. Time of-flight mass spectrometric measurements were performed using a MALDI- TOF/TOF Bruker ULTRAFLEX (mass range: 300–150,000 u) at the Espectrometría de Masas Unit, Universidad Complutense and elemental analyses were determined by the microanalysis facility of Universidad Complutense (Microanálisis Elemental Unit), using a Leco 932 combustion microanalyzer. Elemental analysis confirmed that all final compounds are > 95% purity. Analyses indicated by the symbols of the elements or functions were within ± 0.4 % of the theoretical values.

*6-((bis(Prop-2-yn-1-yl)amino)methyl)benzo[d]thiazol-2-amine (**3**).* 6-(Aminomethyl)benzo[d]thiazol-2-amine (0.12 g, 0.70 mmol) and K_2CO_3 (0.09 g, 0.70 mmol) were mixed in acetonitrile (4 mL) under argon atmosphere and 3-bromoprop-1-yne solution 80 wt. % in toluene (0.10 mL, 0.27 mmol) was added dropwise within 30 min at 0 °C. The mixture was stirred overnight at r.t. and then filtered over a celite pad, which was washed with methanol. The filtrate was concentrated, and the resulting crude product was purified by silica column chromatography (90:1:0.1 dichloromethane/methanol/triethylamine) giving the final compound **3** as a white solid. Yield: 25%. Mp: 148 °C. ^{1}H NMR (250 MHz, $(CD_3)_2CO$) δ 7.65 (d, 1H), 7.41 (d, J = 8.2 Hz, 1H), 7.27 (dd, J = 8.2, 1.1 Hz, 1H), 7.06 (br, 2H), 3.73 (s, 2H), 3.45 (d, J = 2.1 Hz, 4H), 2.80 (t, J = 2.2 Hz, 2H). ^{13}C NMR (63 MHz, $(CD_3)_2CO$) δ 168.2, 153.8, 133.1, 132.9, 128.2, 122.7, 119.6, 80.3, 75.4, 58.1, 42.7. Anal.

Calcd for $C_{14}H_{13}N_3S$: C, C, 65.85; H, 5.13; N, 16.46; S, 12.56. Found: 65.61; H, 5.11; N, 16.40; S, 12.51. HRMS (MALDI-TOF): *m/z*: Calcd for $C_{14}H_{13}N_3S$, 255.0830; found, 255.0829.

*6-((Prop-2-yn-1-ylamino)methyl)benzo[d]thiazol-2-amine (**4**)*. A solution of **11** (0.38 g, 2.56 mmol) in methanol (5 mL), was added dropwise at 0 °C to a stirred solution of propargylamine (0.16 mL, 2.56 mmol) and 4Å molecular sieves in methanol (5 mL). After 2 hours, the pH was adjusted with 10% aqueous HCl to pH = 5, and $NaBH_3CN$ (0.134 g, 2.13 mmol) was added to the reaction mixture. After 12 hours at r.t, methanol was evaporated, and the residue was diluted with ethyl acetate (20 mL). The solution was basified with a saturated solution of $NaHCO_3$ and washed with brine. The organic layers were dried over Na_2SO_4 and concentrated to dryness. The crude product was purified by column chromatography (15:1 dichloromethane/methanol) to give **4** as a yellow solid. Yield 60%. Mp: 130 °C. ^{1}H NMR (250 MHz, CD_3OD) δ 7.56 (d, *J* = 1.4 Hz, 1H), 7.33 (d, *J* = 8.2 Hz, 1H), 7.23 (dd, *J* = 8.2, 1.7 Hz, 1H), 3.84 (s, 2H), 3.35 (d, *J* = 2.5 Hz, 2H), 2.66 (t, *J* = 2.5 Hz, 1H). ^{13}C NMR (63 MHz, CD_3OD) δ 170.2, 152.7, 133.7, 132.4, 128.1, 122.4, 118.8, 82.1, 73.9, 52.8, 37.5. Anal. Calcd for $C_{11}H_{11}N_3S$: C, 60.80; H, 5.10; N, 19.34; S, 14.75. Found: 61.10; H, 5.11; N, 19.40; S, 14.81. HRMS (MALDI-TOF): *m/z*: cal. for $C_{11}H_{11}N_3S$, 217.0674; found, 217.0663.

*6-((Methyl(prop-2-yn-1-yl)amino)methyl)benzo[d]thiazol-2-amine (**5**)*. Formaldehyde 37% in methanol (0.02 g, 0.64 mmol) was added to a solution of **4** (0.07 g, 0.32 mmol) in 10 mL of acetonitrile. After 30 minutes, the solution was acidified to pH = 5 using HCl in methanol and $NaBH_3CN$ (0.04 mg, 0.64 mmol) was added. The reaction was stirred for 3 hours and neutralized with saturated solution of $NaHCO_3$. The aqueous phase was extracted with ethyl acetate (3 x 20 mL) and the crude product was purified by flash column over silica gel with dichloromethane/methanol 15:1 followed by trituration from a mixture of dichloromethane, ethyl ether and petroleum ether. Compound **5** was obtained as a yellow oil. Yield 49 % ^{1}H NMR (250 MHz, CD_3OD) δ 7.55 (d, *J* = 1.3 Hz, 1H), 7.33 (d, *J* = 8.2 Hz, 1H), 7.22 (dd, *J* = 8.2, 1.6 Hz, 1H), 3.62 (d, *J* = 4.4 Hz, 2H), 3.27 (d, *J* = 2.2 Hz, 2H), 2.72 (t, *J* = 2.4 Hz, 1H), 2.33 (s, 3H). ^{13}C NMR (63 MHz, CD_3OD) δ 170.3, 152.9, 132.5, 132.3, 128.9, 123.2, 118.7, 78.9, 75.9, 60.8, 45.8, 42.1. Anal. Calcd for $C_{12}H_{13}N_3S$: C, 62.31; H, 5.66; N, 18.17; S, 13.86. Found: 62.56; H, 5.68; N, 18.24; S, 13.91. HRMS (MALDI-TOF): *m/z*: cal. for $C_{12}H_{13}N_3S$, 231.0830; found, 231.0829.

N-(Prop-2-yn-1-yl)-N-(2-(prop-2-yn-1-ylamino)benzo[d]thiazol-6-yl)acetamide ***(6)***. N-(2-aminobenzo[d]thiazol-6-yl)acetamide (0.30 g, 1.44 mmol) was dissolved in 5 mL of dry DMF and added dropwise to a flask containing NaH 60% dispersion in mineral oil (0.07 g, 2.89 mmol) previously washed with petroleum ether. After stirring the mixture for 2 hours, 3-bromoprop-1-yne solution 80 wt. % in toluene (0.20 g, 1.73 mmol), diluted in 2 mL of dry DMF, was added over 30 minutes with a syringe pump. The reaction mixture was stirred for further 2 hours and then poured into water. The aqueous phase was extracted with ethyl acetate (3 x 50 mL), then the organic phases were concentrated and washed with LiCl aqueous saturated solution (3 x 5 mL) to give **6** as a brown solid. Yield 60 %. Mp: 67 °C. ^{1}H NMR (250 MHz, CD_3OD) δ 7.84 (d, *J* = 2.2 Hz, 1H), 7.51 (dd, *J* = 8.6, 2.3 Hz, 1H), 7.41 (d, *J* = 8.6 Hz, 1H), 4.28 (d, *J* = 2.5 Hz, 2H), 3.76 (d, *J* = 2.6 Hz, 2H), 3.02 (t, *J* = 2.5 Hz, 1H), 2.61 (t, *J* = 2.6 Hz, 1H), 2.13 (s, 3H). ^{13}C NMR (63 MHz, CD_3OD) δ 172.1, 141.3, 135.6, 134.9, 128.4, 121.8, 119.8, 116.2, 80.1, 77.7, 77.5, 73.6, 45.0, 24.2, 21.8. Anal. Calcd for $C_{15}H_{13}N_3OS$: C, 63.58; H, 4.62; N, 14.83; S, 11.32. Found: 63.56; H, 4.68; N, 14.88; S, 11.36. HRMS (MALDI-TOF): *m/z*: cal. for $C_{15}H_{13}N_3OS$, 283.0779; found 283.0777.

*N6-(prop-2-yn-1-yl)-4,5,6,7-tetrahydrobenzo[d]thiazole-2,6-diamine (**7**)*. **14** (0.04 g, 0.30 mmol) and K_2CO_3 (0.07 g, 0.50 mmol) were dissolved in 4 mL of THF. After 1 hour, 3-bromoprop-1-yne solution 80 wt. % in toluene (0.04 g, 0.30 mmol) was added, and the reaction was stirred for further 5 hours. The solvent was evaporated under reduced pressure, the residue was redissolved in ethyl acetate (10 mL) and washed with water (3 x 15 mL). The organic layers were combined, dried over Na_2SO_4 anhydrous and the solvent was evaporated to dryness. Silica gel flash chromatography (9:1 dichloromethane/methanol) afforded **7** as a yellow oil in 8% yield (17 mg). ^{1}H NMR (300 MHz, CD_3OD) δ: 3.49 (dd, *J* = 2.5, 0.9 Hz, 2H), 3.24 – 3.10 (m, 1H), 2.89 – 2.75 (m, 1H), 2.61 (t, *J* = 2.5 Hz, 1H), 2.59 –2.42 (m, 2H), 2.41 – 2.26 (m, 1H), 2.13 – 1.98 (m, 1H), 1.73 – 1.53 (m, 1H). ^{13}C NMR (63 MHz, CD_3OD) δ: 169.7, 144.9, 114.9, 81.8, 73.4, 53.6, 35.8, 29.6, 29.5, 25.6. Anal. Calcd for $C_{10}H_{13}N_3S$: C 57.94, H 6.32, N 20.27, S 15.47. Found: C 58.02, H 6.34, N 20.22, S 15.49. HRMS (MALDI-TOF): *m/z*: cal. for $C_{10}H_{13}N_3S$, 207.0830; found 207.0831

N-(prop-2-yn-1-yl)-N-(2-(prop-2-yn-1-ylamino)-4,5,6,7-tetrahydrobenzo[d]thiazol-6-yl) acetamide ***(8)***. **13** (0.21 g, 1.00 mmol) was dissolved in 5 mL of dry DMF and the solution was added dropwise under argon atmosphere to a flask containing NaH 60% dispersion in mineral oil (0.08 g, 2.00 mmol), previously

washed with petroleum ether. After 1 hour, 3-bromoprop-1-yne solution 80 wt. % in toluene (0.22 g, 1.50 mmol) was added and the reaction stirred overnight at 0 °C under inert atmosphere. The reaction was quenched with water (10 mL), the aqueous phase extracted with ethyl acetate (3 x 15 mL) and the combined organic phases were washed with LiCl aqueous saturated solution (3 x 15 mL) to remove the remaining DMF. The organic phase was dried over anhydrous Na_2SO_4 and concentrated under reduced pressure. The crude was purified by silica gel flash chromatography (9:1 dichloromethane/methanol) to obtain compound **8** (35 mg, 12%). ^{1}H NMR (250 MHz, CD_3OD) δ: 4.22 (dd, *J* = 2.5, 1.6 Hz, 2H), 4.09 – 3.91 (m, 1H), 3.56 (d, *J* = 2.6 Hz, 2H), 3.02 (t, *J* = 2.5 Hz, 1H), 2.83 – 2.72 (m, 1H), 2.70 (t, *J* = 2.6 Hz, 1H), 2.58 – 2.47 (m, 2H), 2.35 (ddt, *J* = 17.0, 8.6, 2.7 Hz, 1H), 2.05– 1.97 (m, 1H), 1.95 (s, 3H), 1.81 – 1.61 (m, 1H). ^{13}C NMR (63 MHz, CD_3OD) δ: 172.8, 136.2, 127.9, 115.2, 80.5, 77.7, 77.0, 73.4, 46.2, 42.7, 35.8, 28.7, 28.0, 22.7, 19.4. Anal. Calcd for $C_{15}H_{17}N_3OS$: C 62.69, H 5.96, N 14.62, S 11.16. Found: C 62.87, H 5.97, N 14.81, S 11.19. HRMS (MALDI-TOF): *m/z*: cal. for $C_{15}H_{17}N_3OS$ 287.1092, found 287.1092.

*2-Aminobenzo[d]thiazole-6-carbonitrile (**9**).* 4-aminobenzonitrile (5.03 g, 42.6 mmol) and NH_4SCN (6.49 g, 85.30 mmol) were dissolved in glacial acetic acid (40 mL) at r.t. After dissolution, a solution of bromine (2.16 mL, 42.32 mmol), diluted in glacial acetic acid (20 mL), was added dropwise over 1 hour using an addition funnel. After stirring at r.t overnight, the mixture was basified with 25% NH_4OH aqueous solution. Compound **9** was obtained as a yellow precipitate, filtered, and dried. Yield 85%. Mp: 205 °C. ^{1}H NMR (250 MHz, $(CD_3)_2SO$) δ 8.19 (d, *J* = 1.7 Hz, 1H), 8.07 (s, 2H), 7.61 (dd, *J* = 8.4, 1.8 Hz, 1H), 7.41 (d, *J* = 8.3 Hz, 1H). ^{13}C NMR (63 MHz, $(CD_3)_2SO$) δ 170.2, 156.7, 131.7, 129.7, 125.2, 119.7, 117.9, 102.0. Anal. Calcd for $C_8H_5N_3S$: C 54.84, H 2.88, N 23.98, S, 18.30. Found (%) C 56.52, H 3.62, N 21.73, S 16.76.

*6-(Aminomethyl)benzo[d]thiazol-2-amine (**10**).* A mixture of **9** (1.00 g, 5.73 mmol) and $NaBH_4$, (0.43 g, 11.40 mmol) in dry THF (20 mL), was refluxed under argon atmosphere. Freshly distilled boron trifluoride diethyl etherate (5.00 mL, 40.51 mmol), diluted in 10 mL of dry THF, was added dropwise to the reaction over 1 hour. After 2 hours, the reaction was quenched with HCl 10%, and basified with 40% w/w aqueous NaOH solution until pH = 11. The mixture was filtered, and the filtrate extracted with dichloromethane (3 x 50 mL). The organic layers were dried over anhydrous Na_2SO_4 and the solvent dried under reduced pressure. Compound **10** was used without any further purification. Yield: 42%. Mp: 152 °C. ^{1}H NMR (250 MHz,

CD_3OD) δ 7.59 (d, 1H), 7.36 (d, *J* = 8.2 Hz, 1H), 7.25 (dd, *J* = 8.2, 1.5 Hz, 1H), 3.83 (s, 2H). ^{13}C NMR (63 MHz, CD_3OD) δ 167.3, 148.6, 138.2, 128.4, 124.8, 120.2, 119.5, 46.5. Anal. Calcd for $C_8H_9N_3S$, C 53.61, H 5.06, N 23.44, S 17.89. Found C 53.53, H 5.05, N 23.47, S 17.86.

*2-Aminobenzo[d]thiazole-6-carbaldehyde (**11**).* A solution of DIBAL-H (5.39 mL, 1.0 M in hexane), diluted in 10 mL of dry dichloromethane, was added dropwise at 0 °C to a solution of **9** (0.31 g, 1.79 mmol) in dry dichloromethane. After 3 hours, the mixture was poured into crushed ice and solution of HCl 6M was added. After 1 hour, the solution was neutralized with saturated solution of $NaHCO_3$ and extracted with ethyl acetate (3 x 50 mL). The organic layers were combined, dried over Na_2SO_4 and concentrated to dryness. The crude was used without further purification. Orange solid. Yield 76%. Mp: 163 °C. ^1H NMR (250 MHz, CD_3OD) δ 9.86 (s, 1H), 8.14 (d, *J* = 1.4 Hz, 1H), 7.79 (dd, *J* = 8.4, 1.7 Hz, 1H), 7.46 (d, *J* = 8.3 Hz, 1H). ^{13}C NMR (63 MHz, CD_3OD) δ 193.0, 173.4, 159.1, 133.2, 132.2, 129.6, 124.5, 119.0. Anal. Calcd for $C_8H_6N_2OS$, C 53.92, H 3.39, N 15.42, S 17.99. Found C 54.02, H 3.40, N 15.42, S 18.01.

*N-(2-aminobenzo[d]thiazol-6-yl)acetamide (**12**).* N-(4-aminophenyl)acetamide (1.00 g, 6.66 mmol) and NH_4SCN (1.13 g, 13.32 mmol) were dissolved in glacial acetic acid (20 mL) at r.t. After dissolution, a solution of bromine (0.93 g, 6.66 mmol), diluted in glacial acetic acid (5 mL), was added dropwise over 30 minutes using a syringe pump. After stirring at r.t overnight, the mixture was basified with NH_4OH 25% aqueous solution and extracted with ethyl acetate (3 x 50 mL), the organic layers collected and dried over Na_2SO_4 anhydrous. Final compound **12** was obtained as a yellowish solid by trituration from THF. Yield 55%. Mp: decomposes above 220 °C. ^1H NMR (250 MHz, CD_3OD) δ 7.96 (d, *J* = 1.8 Hz, 1H), 7.29 (d, *J* = 8.6 Hz, 1H), 7.23 (dd, *J* = 8.7, 1.9 Hz, 1H), 2.11 (s, 3H). ^{13}C NMR (63 MHz, CD_3OD) δ 171.8, 169.6, 150.0, 134.5, 132.5, 120.1, 118.8, 114.3, 23.9. Anal. Calcd for $C_9H_9N_3OS$, C 52.16, H 4.38, N 20.27, S 15.47. Found C 52.85, H 4.35, N 20.12, S 15.47.

*N-(2-amino-4,5,6,7-tetrahydrobenzo[d]thiazol-6-yl) acetamide (**13**).* N-(4-oxocyclohexyl)acetamide (0.31 g, 2.00 mmol), NH_4SCN (0.288 g, 4.00 mmol) and I_2 (0.50 g, 2.00 mmol) were dissolved in 2 mL of methanol in a microwave tube. The reaction mixture was heated under microwave irradiation for 10 minutes at 100 °C (200 W). The mixture was cooled to r.t. and methanol was added until a yellow precipitate was formed. The solid was filtrated and used without further purification. Yield 66 %. Mp: 247-250 °C. ^1H NMR

(250 MHz, CD_3OD) δ: 4.26 – 4.10 (m, 1H), 2.87 (dd, *J* = 16.2, 5.4 Hz, 1H), 2.65 (td, *J* = 7.0, 1.7 Hz, 2H), 2.45 (ddt, *J* = 16.1, 7.9, 2.3 Hz, 1H), 2.13 – 1.97 (m, 1H), 1.96 (s, 3H), 1.93 – 1.77 (m, 1H). ^{13}C NMR (63 MHz, CD_3OD) δ: 173.0, 171.3, 134.2, 114.9, 46.1, 29.1, 27.8, 22.6, 22.1. Anal. Calcd for $C_9H_{13}N_3OS$, C 51.16, H 6.20, N 19.89, S15.17. Found C 51.28, H 6.23, N 19.91, S 15.19.

*4,5,6,7-tetrahydrobenzo[d]thiazole-2,6-diamine (**14**).* Compound **13** (0.10 g, 0.50 mmol) was dissolved in 4 mL of HBr solution 6M and the mixture stirred at 110 °C. After 16 hours the solvent was concentrated under reduced pressure and the mixture was neutralized with saturated solution of Na_2CO_3. The obtained precipitate washed several times with acetone to give compound **14** to be used without further purification. Yield 96 %. Mp: 217. ^1H NMR (250 MHz, CD_3OD) δ: 3.81 – 3.64 (m, 1H), 3.13 – 2.97 (m, 1H), 2.82 – 2.59 (m, 3H), 2.35 – 2.17 (m, 1H), 2.12 – 1.92 (m, 1H). ^{13}C NMR (63 MHz, CD_3OD) δ: 171.5, 134.1, 112.9, 47.6, 27.7, 26.4, 21.7. Anal. Calcd for $C_7H_{11}N_3S$, C 49.68, H 6.55, N 24.83, S 18.94. Found C 49.87, H 6.56, N 24.73, S 18.99.

Molecular modelling studies of *h*MAO inhibition. Molecular modelling studies were carried out by using the Schrödinger Suite version 2018-1.

Protein and ligands preparation. For *in silico* simulations, the crystallographic structures 2BXS[3] and 6FW0[4] were downloaded from the Protein Data Bank (PDB), for *h*MAO-A and *h*MAO-B, respectively. Thus, PDB structures were submitted to the Protein Preparation Wizard tool using OPLS_2005 as force field.[5] During this process, (i) hydrogen atoms were added, (ii) partial charges were assigned, (iii) missing atoms, side chains and loops were built, (iv) all water molecules were deleted, (v) co-crystallized ligands were removed, and (vi) FAD bonding orders were fixed. The 3D structures of all compounds were drawn by means Maestro GUI, while LigPrep module was used for modelling and calculating their protonation state at pH 7.4 and any enantiomers for each chirality center.[6] All ligands were finally optimized using OPLS_2005 force field.

Docking simulation. For docking studies, the grid box for active sites was built by centering on the FAD N5 atom, by evaluating a volume of about 64.000 Å^3. The software Glide version *7.8* was used to generate the docking poses, by applying the Glide Extra-Precision (XP) protocol. The ligand structural flexibility was taken

into account and ten poses per ligand were generated. Finally, the default docking scoring function was used for selecting the best binding mode for each ligand.

Molecular dynamics. **5**, **4**, **6** and **8** 's best docking complexes with *h*MAO-A have been subjected to MD simulation (MDs). In particular, the Desmond package was used for MDs, employing OPLS_2005 as force field. An orthorhombic water box was built for the system solvation, by ensuring a buffer distance of approximately 10 Å between each box side and the complex atoms. MD suitable systems were generated by applying the SPC explicit solvent model and neutralized by adding Na^+ counterions. Thus, solvated systems were equilibrated through the application of default Desmond protocol. All MDs were carried out at 300 K, up to 200 *ns*, considering an integration time step equal to 2 *fs*. MD frames were sampled at regular time intervals equal to 200 *ps*. Finally, the Simulation Interaction Diagram of Desmond was used to analyze all MD runs.

PAMPA assay. PAMPA assay was carried out in a coated 96-well membrane filter according to the protocol used in [7]. The tested compounds (**3-8**) were dissolved first in DMSO and then diluted with PBS pH 7.4 to reach the final concentrations of 50-500 µM in the donor well. The final concentration of DMSO did not exceed 0.5% (v/v) in the donor solution. 300 µl of the donor solution (V_D) was added to the donor wells and the donor filter plate was carefully put on the acceptor plate so that the coated membrane was "in touch" with both donor solution and acceptor buffer. The concentration of the tested compounds in both donor and the acceptor wells were assessed after 3, 4, 5 and 6 hours of incubation respectively in quadruplicate using the UV plate reader Synergy HT (Biotek, USA) at the maximum absorption wavelength of each compound (n = 3 for reference compounds, n = 2 for novel compounds).

Evaluation of Human Monoamine Oxidase (*h*MAO) Inhibitory Activity. The *h*MAO inhibitory activity was assessed in microsomal MAO isoforms prepared according to the experimental protocol described elsewhere.[8] The appropriate amounts of *h*MAO-A and *h*MAO-B were adjusted to obtain, in our experimental conditions, the same maximum velocity (V_{max} = 50 $pmol.min^{-1}$) for both isoforms (*h*MAO-A: 3 $ng.\mu L^{-1}$; *h*MAO-B: 12 $ng.\mu L^{-1}$). All assays were performed in sodium phosphate buffer solution 50 mM pH 7.4.

Compounds **3-8**, **1** or **2** were pre-incubated at 37 ºC for 10 min in the presence of kynuramine (K_m *h*MAO-A = 20 µM; K_m *h*MAO-B = 20 µM; final concentration: 2×K_m) in 96-well microplates (BRANDplates, pureGrade™, BRAND GMBH, Wertheim, Germany). Then, the reaction was started with the addition of *h*MAO-A or *h*MAO-B. Initial velocities were determined spectrophotometrically in a microplate reader (BioTek Synergy HT from BioTek Instruments, Winooski, VT, USA) at 37 °C by measuring the formation of 4-hydroxyquinoline at 316 nm, over a period of at least 30 min (interval of 1 min). Data were analysed using GraphPad PRISM version 6 for Windows (GraphPad Software®, San Diego, CA, USA). The initial velocities, obtained from the linear phase of product formation, were normalized and plotted against the respective inhibitor concentration. IC_{50} values were obtained from dose-response curves and were expressed as mean ± standard deviation. IC_{50} values were determined from at least three independent experiments, each performed in triplicate.

Cell viability in lymphoblasts. Lymphoblast cell lines from one ALS patient with mutant SOD1 (mutSOD1) and its sex an age-matched control were obtained from a cell line repository of Coriell Institute for Medical Research, USA (www.coriell.org). The two lymphoblast cell lines were both from 46 years-old-men. Lymphoblasts were maintained at a density of 400,000–1200,000 cells/mL and cultured in RPMI-1640 medium supplemented with 2 g/L sodium bicarbonate, 15% (v/v) FBS and 0.5% of penicillin-streptomycin plus amphotericin B (v/v) in T25 or T75 flasks, in upright position, at 37°C in a humidified atmosphere of 5% CO_2. Before all experiments, lymphoblasts were plated at 400,000 or 700,000 cells/mL in RPMI medium and incubated for 48 h or 24 h, respectively, to secure a cell density of around 1000,000 cell/mL in each assay. To determine the cell viability, cells were seeded at a concentration of 160,000 cells in 96-well plates, with a final volume of 50 µL per well and treated with three concentrations for each compound (1,10,100 µM), references and mix (rasagiline + riluzole). After 24h, it was added 50 µL of resazurin solution at 20 µg/mL and incubated for 2 h at 37 °C and 5% CO_2. The reduction of resazurin to resorufin was measured fluorometrically at 540 nm excitation and 590 nm emission in the Biotek Cytation 3 spectrophotometer (BioTek Instruments Inc., USA).

Antioxidant activity in human lymphoblasts. LH5 and LPS5 lymphoblast-derived cell lines (corresponding to a 46 year-old male ALS patient with mutant SOD1 and its sex an age-matched control, respectively) were seeded in 96-well plates, at $0.8x10^6$ cells/mL, in 100 µL RPMI-1640 medium, supplemented with 2 g/L sodium bicarbonate, 15% (v/v) FBS and 0.5% penicillin-streptomycin plus amphotericin B (v/v), at 37 °C, in a humidified atmosphere of 5% CO_2, for 24h before the experiments. Then, cells were pretreated with 10 µM of compounds **1**, **2**, **1+2**, **5** and **6**, or 0.03% DMSO (as control vehicle) for 21h. Then, the lymphoblasts were exposed to 10 µM menadione (2-methyl-1,4-naphthoquinone) or 0.04% ethanol (as the control vehicle for menadione) for further 3h, at 37°C, in 5% CO_2. Finally, 100 µL resazurin solution (20 µg/mL) were added and the cells incubated for 2h at 37 °C, in 5% CO_2. The reduction of resazurin to resorufin was measured fluorometrically using an excitation wavelength of 540 nm and an emission wavelength of 590 nm, in a Biotek Cytation 3 spectrophotometer (BioTek Instruments Inc., USA). Results were calculated as percentage of control.

Neuroprotective effect on glutamate-mediated excitotoxicity in CGNs. Primary cerebellar granule cells were prepared according to [9]. After 8 days *in vitro*, differentiated neurons were shifted to serum free BME medium containing 25 mmol/L KCl and pretreated (2h) with increasing concentrations of **1**, **2**, **5**, **6** (1, 10, 25, µM) as well as 1:1 combination of **1** and **2** in the presence of glutamate 100 µM for 24h. Then, cell viability was evaluated through the MTT assay and absorbance was read at 570 nm in a multiplate spectophotometric reader (Bio-Rad).

Immunomodulation in N9 cell line. N9 microglial cells were plated at a density of 1.2×10^5 cell/35mm Ø dish in serum free DMEM High Glucose (Life Technologies) and pretreated (2h) with increasing concentrations of **1**, **2**, **5**, **6** (1, 10, µM) as well as 1:1 combination of **1**+**2** in the presence of LPS 100 ng/ml for 24 h. The microglial phenotype was evaluated through Western blot analysis of the M1-iNOS (inducible Nitric Oxide Synthase) and M2-TREM2 (Triggering Receptor Expressed on Myeloid cells 2) markers, following the protocol reported in [9].

Antiapoptotic effects in N9 cell line. N9 microglial cells were plated at a density of 1.2 x 10^5 cell/35mm Ø dish in serum free DMEM High Glucose (Life Technologies) and pretreated (2h) with increasing concentrations of **1**, **2**, **5**, **6** (1 and 10 μM) as well as 1:1 combination of **1**+**2** in the presence of LPS 100 ng/ml for 24 h. The programmed cell death was evaluated through Western blot analysis of the antiapoptotic marker Bcl-2 (B-cell lymphoma 2).

Molecular Docking at CK-1δ. The CK-1δ three-dimensional structure (PDB ID 5OKT) has been curated using the MOE structure preparation tool and the ionization state of charged residues has been predicted exploiting the MOE-Protonate-3D tool.[3,4] The molecular docking protocol GOLD and the GoldScore scoring function have been used to predict the ligand accommodation states.[5] The CK-1δ active site has been defined around the coordinates of ligand 9XK center of mass, using a searching sphere of 10 Å radius. Individual electrostatic and hydrophobic interactions, hereinafter identified as IE_{ele} and IE_{hyd}, have been computed between each ligand pose and all protein residues involved in binding. Both these contributions have been computed using MOE and, in particular, IE_{ele} has been calculated as nonbonded electrostatic interactions energy term of the force field, so it's expressed in kcal/mol. IE_{hyd} has instead been computed as contact hydrophobic surfaces and are associated to an adimensional score (the higher the better). The data obtained by this analysis have been reported in a plot, defined Interaction Energy Fingerprints (IEFs), representing residues (x-axis) in the form of equally sized rectangles rendered according to a colorimetric scale. As regards IE_{ele}, colors from blue to red represent energy values ranging from negative to positive values; for IE_{hyd}, colors from white to dark green depict scores going from 0 to positive values. Molecular docking studies have elucidated how the substitution of riluzole (**1**) trifluoromethyl group with propargylic derivatives (compounds **3-5**) is geometrically tolerated, since it's not disrupting the key polar interactions anchoring the ligand to the critical CK-1δ Hinge region (residues E83 to G86 in the IE_{ele} and IE_{hyd} plots). However, the positive charge presents in all riluzole-rasagiline hybrids (compounds **3-5** have been modelled in this study in the physiologically prominent ionized form) contributes to destabilizing the complexes, mediating repulsive electrostatic interactions with the catalytic residue K38 (IE_{ele} plots).

CK-1δ activity assay. Compounds were evaluated towards CK-1δ (Merck Millipore, recombinant human, amino acids 1‑294, with N-terminal GST-tagged) with the KinaseGloR luminescence assay (Promega)

following procedures reported in literature.[10] In detail, luminescent assays were performed in black 96-well plates, using a buffer composed of: 50 mM HEPES (pH 7.5), 1 mM EDTA, 1 mM EGTA, and 15 mM magnesium acetate. Compound CR8 (IC_{50} = 0.4 μM) was used as a positive control[11] while DMSO/buffer solution was used as a negative control. In a typical assay, 10 μL of inhibitor solution (obtained using a 10 mM DMSO solution diluted in assay buffer to 160 μM that corresponds to a final concentration of 40 μM in the assay) and 10 μL (16 ng of CK1δ) of enzyme solution were added to the well, followed by 20 μL of assay buffer containing 0.1% casein substrate and 4 μM ATP. The final DMSO concentration in the reaction mixture did not exceed 1-2%. After 60 min of incubation at 30 °C for CK1δ the enzymatic reactions were stopped with 40 μL of Kinase-Glo reagent (Promega). Luminescence signal (relative light unit,RLU) was recorded after 10 min at 25 °C using Tecan Infinite M100. The activity is proportional to the difference of the total and consumed ATP. The residual activities of the enzyme were calculated on the basis of maximal activities measured in the absence of inhibitor. Data were analyzed using Excel and percentages of enzyme residual activity are reported as means ± standard deviation of two independent experiments performed in technical duplicate.

Bibliography

(1) Wager, T. T.; Hou, X.; Verhoest, P. R.; Villalobos, A. Moving beyond rules: the development of a central nervous system multiparameter optimization (CNS MPO) approach to enable alignment of druglike properties. *ACS Chem Neurosci* **2010**, *1* (6), 435-449. DOI: 10.1021/cn100008c.

(2) Csizmadia, P. MarvinSketch and MarvinView: molecule applets for the World Wide Web. **1999**. Lagorce, D.; Bouslama, L.; Becot, J.; Miteva, M. A.; Villoutreix, B. O. FAF-Drugs4: free ADME-tox filtering computations for chemical biology and early stages drug discovery. *Bioinformatics* **2017**, *33* (22), 3658-3660. DOI: 10.1093/bioinformatics/btx491.

(3) De Colibus, L.; Li, M.; Binda, C.; Lustig, A.; Edmondson, D. E.; Mattevi, A. Three-dimensional structure of human monoamine oxidase A (MAO A): relation to the structures of rat MAO A and human MAO B. *Proc Natl Acad Sci U S A* **2005**, *102* (36), 12684-12689. DOI: 10.1073/pnas.0505975102 From NLM.

(4) Reis, J.; Manzella, N.; Cagide, F.; Mialet-Perez, J.; Uriarte, E.; Parini, A.; Borges, F.; Binda, C. Tight-Binding Inhibition of Human Monoamine Oxidase B by Chromone Analogs: A Kinetic, Crystallographic, and Biological Analysis. *J Med Chem* **2018**, *61* (9), 4203-4212. DOI: 10.1021/acs.jmedchem.8b00357.

(5) Shivakumar, D.; Harder, E.; Damm, W.; Friesner, R. A.; Sherman, W. Improving the Prediction of Absolute Solvation Free Energies Using the Next Generation OPLS Force Field. *J Chem Theory Comput* **2012**, *8* (8), 2553-2558. DOI: 10.1021/ct300203w.

(6) LigPrep; Maestro; Suites, S.; Schrödinger, L. N. Y., NY, USA. 2018.

(7) Perone, R.; Albertini, C.; Uliassi, E.; Di Pietri, F.; de Sena Murteira Pinheiro, P.; Petralla, S.; Rizzardi, N.; Fato, R.; Pulkrábková, L.; Soukup, O.; et al. Turning donepezil in a multi-target-directed ligand through a rationale merging strategy. *ChemMedChem* **2020**. DOI: 10.1002/cmdc.202000484.

(8) Hagenow, S.; Stasiak, A.; Ramsay, R. R.; Stark, H. Ciproxifan, a histamine H 3 receptor antagonist, reversibly inhibits monoamine oxidase A and B. *Scientific reports* **2017**, *7* (1), 1-6. Chavarria, D.; Cagide, F.; Pinto, M.; Gomes, L. R.; Low, J. N.; Borges, F. Development of piperic acid-based monoamine oxidase inhibitors: Synthesis, structural characterization and biological evaluation. *Journal of Molecular Structure* **2019**, *1182*, 298-307.

(9) Uliassi, E.; Peña-Altamira, L. E.; Morales, A. V.; Massenzio, F.; Petralla, S.; Rossi, M.; Roberti, M.; Martinez Gonzalez, L.; Martinez, A.; Monti, B.; et al. A Focused Library of Psychotropic Analogues with Neuroprotective and Neuroregenerative Potential. *ACS Chem Neurosci* **2018**. DOI: 10.1021/acschemneuro.8b00242.

(10) Salado, I. G.; Redondo, M.; Bello, M. L.; Perez, C.; Liachko, N. F.; Kraemer, B. C.; Miguel, L.; Lecourtois, M.; Gil, C.; Martinez, A.; et al. Protein kinase CK-1 inhibitors as new potential drugs for amyotrophic lateral sclerosis. *J Med Chem* **2014**, *57* (6), 2755-2772. DOI: 10.1021/jm500065f. Grieco, I.; Bissaro, M.; Tiz, D. B.; Perez, D. I.; Perez, C.; Martinez, A.; Redenti, S.; Mariotto, E.; Bortolozzi, R.; Viola, G.; et al. Developing novel classes of protein kinase CK1δ inhibitors by fusing [1,2,4]triazole with different bicyclic heteroaromatic systems. *Eur J Med Chem* **2021**, *216*, 113331. DOI: 10.1016/j.ejmech.2021.113331 From NLM.

(11) Bettayeb, K.; Oumata, N.; Echalier, A.; Ferandin, Y.; Endicott, J. A.; Galons, H.; Meijer, L. CR8, a potent and selective, roscovitine-derived inhibitor of cyclin-dependent kinases. *Oncogene* **2008**, *27* (44), 5797-5807. DOI: 10.1038/onc.2008.191 From NLM.
